# Exclusive breastfeeding promotion and neuropsychological outcomes in 5-8 year old children from Uganda and Burkina Faso: Results from the PROMISE EBF cluster randomized trial

**DOI:** 10.1371/journal.pone.0191001

**Published:** 2018-02-23

**Authors:** James K. Tumwine, Victoria Nankabirwa, Hama Abdoulaye Diallo, Ingunn Marie Stadskleiv Engebretsen, Grace Ndeezi, Paul Bangirana, Anselme Simeon Sanou, Espérance Kashala-Abotnes, Michael Boivin, Bruno Giordani, Irene Bircow Elgen, Penny Holding, Angelina Kakooza-Mwesige, Vilde Skylstad, Joyce Nalugya, Thorkild Tylleskar, Nicolas Meda

**Affiliations:** 1 Department of Paediatrics and Child Health, School of Medicine, College of Health Sciences, Makerere University, Kampala, Uganda; 2 Centre for Intervention Science in Maternal and Child Health (CISMAC), Centre for International health, Department of Global Public Health and Primary Health Care, Faculty of Medicine and Odontology, University of Bergen, Bergen, Norway; 3 School of Public Health, College of Health Sciences, Makerere University, Kampala, Uganda; 4 Centre MURAZ, Ministry of Health, Bobo-Dioulasso, Burkina Faso; 5 Centre for International Health, Department of Global Public Health and Primary Health Care, Faculty of Medicine and Odontology, University of Bergen, Bergen, Norway; 6 Department of Psychiatry, School of Medicine, College of Health Sciences, Makerere University, Kampala, Uganda; 7 Department of Psychiatry, Michigan State University, East Lansing, Michigan, United States of America; 8 Division of Neuropsychology, Department of Psychiatry, University of Michigan, Ann Arbor, Michigan, United States of America; 9 Department of Child and Adolescent Psychiatry, Haukeland University Hospital, Bergen, Norway; 10 Identitea, Nairobi, Kenya; TNO, NETHERLANDS

## Abstract

**Background:**

The beneficial effects from exclusive breastfeeding (EBF) have been widely acknowledged. We assessed the effect of exclusive breastfeeding promotion by peer counsellors in Uganda and Burkina Faso, on cognitive abilities, social emotional development, school performance and linear growth among 5–8 years old children.

**Methods:**

Children in the PROMISE EBF trial (2006–2008) were re-enrolled in the follow-up PROMISE Saving Brains (SB) study (2013–2015). Caretaker interviews captured sociodemographic characteristics and social emotional development using the parent version of the Strengths and Difficulties Questionnaire (SDQ). Overall cognition and working memory were assessed using the Kaufman Assessment Battery for Children, second edition (KABC2), cognitive flexibility was measured with the Child Category Test (CCT), and attention with the Test of Variables of Attention (T.O.V.A), while school performance was measured by a standardized test on arithmetic and reading. Country-pooled, age adjusted z-scores from each of the above outcomes were entered into a linear regression model controlling for confounders.

**Results:**

The number of children re-enrolled in the intervention and control arms were: 274/396 (69.2%) and 256/369 (69.4%) in Uganda and 265/392 (67.6%) and 288/402 (71.6%) in Burkina Faso. Assessment of cognitive ability showed small and no significant differences, of which general cognition (z-scores, 95% CI) showed the largest mean difference: -0.17 (-0.40; 0.05). Social emotional symptoms were similar across arms. There were no differences in school performance or linear growth for age detected.

**Conclusion:**

Peer promotion for exclusive breastfeeding in Burkina Faso and Uganda was not associated with differences at 5–8 years of age in a range of measures of child development: cognitive abilities, emotion-behaviour-social symptoms or linear growth. This study from sub Saharan Africa did not reconfirm findings elsewhere that have shown an association between exclusive breastfeeding and cognitive performance. This might be due to a number of methodological limitations inherent in the current study. For example since the majority of the children were breastfed, the benefits of the intervention could have been diluted. Other factors such as the mental and HIV status of the mothers (which were not assessed in the current study) could have affected our results. Hence regarding the effect of exclusive breastfeeding on measures of child neurocognitive development in sub Saharan Africa, the jury is still out.

**Trial registration:**

ClinicalTrials.gov NCT01882335

## Introduction

Infant and child health and development have received increasing attention in the sustainable development goals (SDGs). In light of this there is a need to find ways to deliver interventions which promote healthy nutrition and child development [1, 2]. Exclusive breastfeeding (EBF) promotion has been recommended as one of the key interventions for a good start in childhood [1]. Even if breastfeeding has been the norm in sub-Saharan Africa, exclusive breastfeeding has not been common [1], and recent studies elude to country variations and lack of data for many sub-Saharan countries [2]. The average exclusive breastfeeding prevalence in the sub-Saharan countries with high diarrhea prevalence based on the last DHS reports was 34%, where a prevalence of 25% and 63% were seen in Burkina Faso and Uganda, respectively [2].

Over the last decade a causal relationship between breastfeeding and cognitive performance in childhood has been debated and a recent meta-analysis supported such a relationship [3]. The authors of the review described a causal relationship between breastfeeding and intelligence and discussed the role of publication- and selection bias, maternal intelligence, home environment and stimulation. That was an update of prior work done in 2007 [4] and 2013 [5] also concluding that breastfeeding had long term positive effects on intelligence, school performance and adult income. The review from 2015 included eighteen cohort studies of which 16 were from high-income countries [3]. Similar findings have recently been supported among toddlers in a cohort from Singapore [6] and from a prospective cohort studying intelligence, educational attainment and income in adulthood from Brazil [7]. On the other hand, another review, consisting, mostly, of studies from high income countries, argued that the observed effect is mainly due to confounding and suggests alternative study design and a better control for confounders [8]. Nonetheless, there is a paucity of information from sub-Saharan Africa, especially from randomised controlled trials promoting EBF. PROBIT, the largest EBF promotion trial, evaluated clinic based breastfeeding promotion in Belarus and found the intervention was positively related to cognitive development [9, 10].

Generally, breastfeeding follow-up studies done in LMICs have reported on morbidity and mortality outcomes rather than cognitive performance indicators. Recently, a systematic review was published on differential infant feeding modalities on infection-related and all-cause mortality for children aged 0–23 months in LMICs suggesting an inverse relationship in risk for mortality with exclusive breastfeeding [11]. One study on breastfeeding and school achievement in five adult cohorts, none from Africa, found varying association between the five countries. This illustrates how important context is for this type of assessment. Further, the authors attributed the differences to confounding factors, particularly gender, maternal age, schooling, smoking during pregnancy, birthweight, socio-economy and father’s occupation and in some sites skin colour and urbanity [5]. Maternal schooling was the strongest positive predictor for school achievement and an observed effect between infant feeding and school achievement changed or disappeared on adjustment for confounding variables.

The PROMISE Saving Brains (PROMISE SB) is a follow-up study of children from the cohorts in Burkina Faso and Uganda enrolled in the PROMISE EBF cluster randomised trial [12] that investigated cognitive and behaviour development and linear growth at 5–8 years of age. The objective of the PROMISE SB study was to assess the effect of peer counselling for EBF in the first six months of life on cognitive abilities, social emotional development, school performance and linear growth among 5–8 years old children in Uganda and Burkina Faso. During the EBF trial, mother-infant pairs in the intervention clusters received peer support for breastfeeding, and the primary endpoints of the trial were the effect of peer counselling on exclusive breastfeeding and diarrhoea prevalence ratios [12]. Results from this trial suggested that peer counsellors effectively contributed to promotion of exclusive breastfeeding. The 7-day recall showed an improvement in the intervention arm in Burkina Faso and Uganda, respectively, of 77% versus 23% (prevalence rate, 95% confidence interval of 3.3, 2.1–5.0) and 77% versus 34% (2.3, 2.0–2.7). The follow-up study is justified by the need to understand the relationship between EBF promotion and long term outcomes in sub-Sharan Africa. To the best of our knowledge, none of the studies on breastfeeding promotion conducted in sub-Saharan Africa have measured the long term effects on cognitive abilities, behavioural symptoms, school performance or linear growth in school age children.

## Methods

### Site, population, randomisation and sampling

This study was conducted among participants of the PROMISE-EBF trial [12] which was a community-based, cluster-randomized trial promoting peer counselling for exclusive breastfeeding (EBF) in the first six months of life. The PROMISE-EBF trial was conducted from 2006 to 2008 in Uganda, Burkina Faso, Zambia and South Africa. Burkina Faso and Uganda were chosen for follow-up as these two countries had the largest, and similar effects from peer support, increasing the EBF prevalence rates around two-fold at 12 weeks and more than three-fold around 24 weeks [12].

In Burkina Faso, the study site was located in and around Banfora, with a population of 94,000 people and lying 85 kilometres south west of Bobo-Dioulasso, the second largest city in the country, with an agricultural and gold mining base to the economy. The Ugandan site was Mbale district and included both urban Mbale Municipality as well as the surrounding rural areas. This district borders Kenya and is characterised by petty trading, small scale industries and peasant farming.

The cluster randomised PROMISE EBF trial [12] was stratified by urban/rural status in Uganda, resulting in 6 urban and 18 rural clusters closely mirroring the population distribution at that time which was approximately 20% urban and 80% rural. In Burkina Faso, the 24 clusters were predominantly rural. In both countries, clusters were selected in close collaboration with the community leaders. Care was taken to allow for ‘corridors’ between selected clusters to avoid potential contamination across clusters.

#### Inclusion and exclusion criteria

Inclusion into the PROMISE EBF trial was a two-stage process involving first the pregnant woman (pre-inclusion) and then the infant (inclusion). The pre-inclusion criteria were that the woman resided in the selected cluster; was at least 7 months or visibly pregnant and had no intention of leaving the study area for at least one year. Following delivery, a pre-included mother-infant pair was included if it was a single birth with no severe malformation that could interfere with breastfeeding. Exclusion criteria included any condition which could hamper informed consent in the mother and intention to replacement feed. In Burkina Faso, the mother-infant pairs included for data collection for the PROMISE EBF trial were randomly done (see page 8 web-appendix [12]) in the intervention clusters as the mothers receiving the intervention exceeded the sample size needed. In Uganda, all mothers in the intervention clusters receiving the intervention were approached for data collection.

#### Intervention

Enrolled mothers in the intervention clusters were offered at least five home-based breastfeeding visits from peer counsellors, starting from the third trimester [13]. These peer counsellors were selected from the same communities as the study participants and trained for one week. The course material, tailored to the local circumstances, was based on the WHO courses: Breastfeeding counselling: a training course, and HIV and infant feeding counselling: a training course [14, 15]. The control group received the standard of care within the respective countries. This included common health information the women were given at antenatal clinics, during birth and at follow-up visits. We have reported details of the feeding intervention, results [16, 17] and follow-up outcomes such as cost-effectiveness [18], diarrhoea [12], growth [19, 20] and mortality [21, 22] up to 24 weeks; and oral health [23], growth and mortality [24, 25] up to 5 years from the PROMISE EBF. At the time of the intervention, the standard-of-care was limited HIV prevention-of-mother-to child care services where very few were tested, got a result and individually targeted infant feeding advice.

#### Re-enrolment

The follow up for the current PROMISE SB study was conducted between 2013 and 2015 when the children were between 5 and 8 years of age. Of the 794 mother-infant pairs enrolled in the PROMISE EBF cohort in Burkina Faso and 765 pairs in Uganda, 553 (69.6%) and 530 (69.3%) were included in this study in Burkina Faso and Uganda, respectively. The number of children re-enrolled in the intervention and control arms were: 275/396 (69.4%) and 256/369 (69.4%) in Uganda and 265/392 (63.3%) and 288/402 (68.2%) in Burkina Faso. 1 person in Uganda was excluded from analysis.

**Fig 1:**
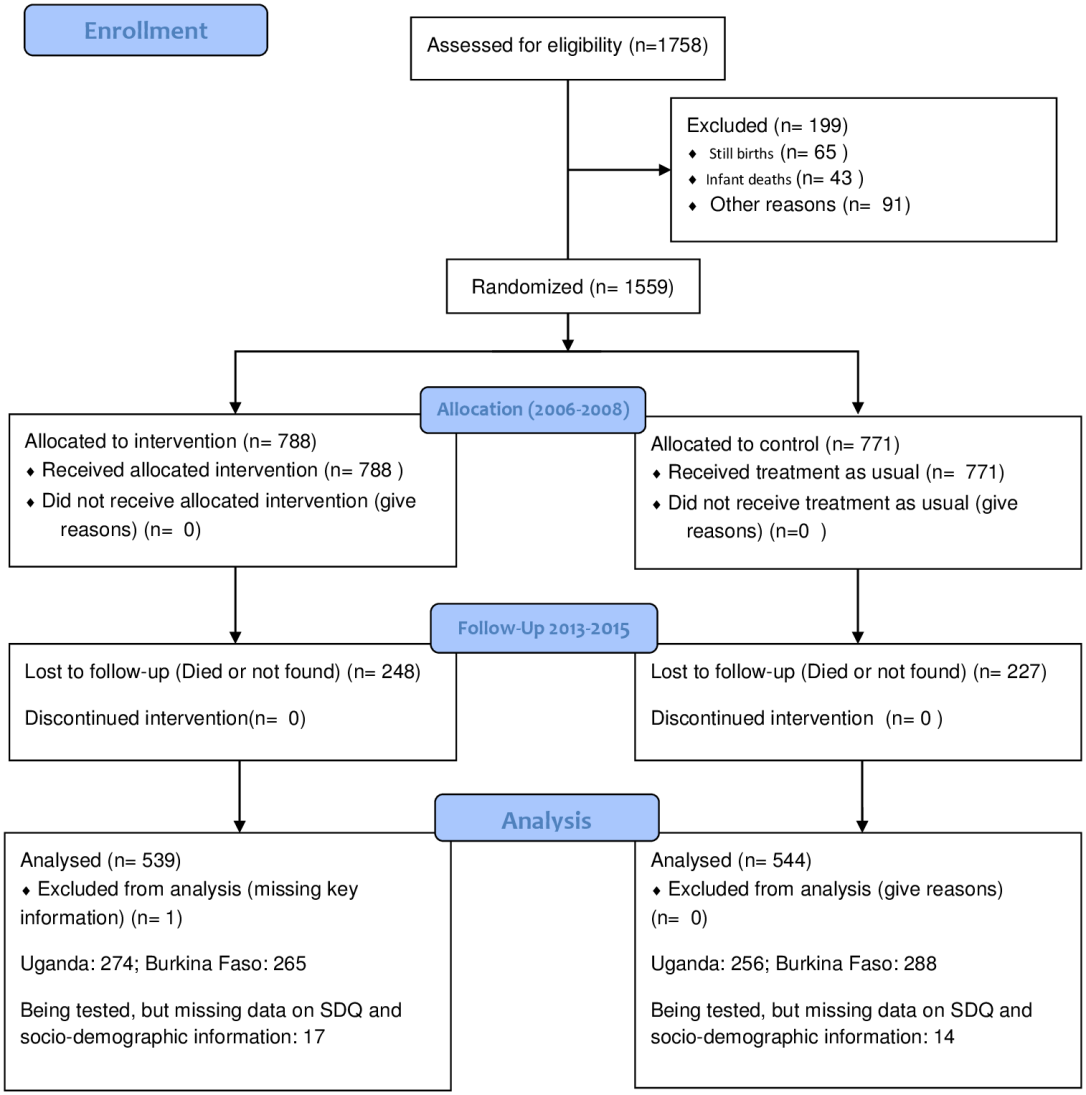
Study profile of the Saving Brains study for Uganda and Burkina Faso.

In Burkina Faso, of the 241 children who were not found or had died, 4 were confirmed lost to follow-up, and for 1 child, the parents withheld consent. The corresponding numbers in Uganda were 168 children not found and 66 children had died. The trial profile is given, Fig 1. Not all consenting participants managed or agreed to participate in all activities, and the respective tables indicate the numbers for each sub-scale.

### Outcome measures

The study outcomes included cognitive abilities, behavioural symptoms, school performance, linear growth and are described below.

#### Cognitive abilities assessed in the study

Cognition: Kaufman Assessment Battery for Children^®^, second edition (KABC-II) [26] is a widely used measure for cognition in sub-Saharan Africa and has been validated in Ugandan children to assess Sequential Processing (short term memory), Simultaneous Processing (visual processing), Learning (long term storage and retrieval) and Planning or problem solving (fluid reasoning). Summation of these four outcomes gives the Mental Processing Index (MPI), a measure of overall cognitive ability. Ten sub-scales were used for 7 years and above, and eleven for younger than seven years. The Rebus-subscale was used in Uganda, but not in Burkina Faso. In this study the KABC-II was used to assess overall cognitive ability using the MPI and Working Memory using the Sequential Processing score. The KABC-II has been used in various African rural populations [27–29] and requires minimal use of language during its administration. Even if not normed for the populations from Uganda or Burkina Faso it was considered as the most favourable cognitive performance test for the purpose. The testing took about 2–2.5 hours.

Attention was measured using the Test of Variables of Attention (T.O.V.A.^®^) [30]. The TOVA is a computer assisted continuous performance measure of attention. The child is required to press a switch immediately as the target stimulus appears on the screen and refrain from pressing when the non-target appears. It measures the child’s response time, impulsivity (pressing when the non-target appears), inattention (failing to press when the target appears) and D Prime (a measure of overall attention ability). Impulsivity was used as a proxy measure of inhibition in the present study. Following instructions and practice trials, TOVA takes about 11 minutes for children younger than 5 and a half years of age and 22 minutes to administer for children 5.5 years and older.

Cognitive flexibility was assessed using the children’s category test (CCT ^®^) [31]. CCT is a measure of nonverbal learning and memory, concept formation, and problem-solving abilities. It is composed of two levels and we used Level 1 for children aged 5 to 8 years. The testing took 10–20 minutes.

Social Emotional Development: This was assessed using the Strengths and Difficulties questionnaires (SDQ) (sdqinfo.org), parent version age range 4–17 years in French and English. The SDQ is a widely used screening tool for mental health symptoms in children.

It is composed of 25 items about the child’s behaviour, rated on a 3-point Likert scale (0 = Not true, 1 = somewhat true, 2 = certainly true) which the caregiver responds to. These items are summarized into five scales; 1) emotional symptoms, 2) conduct problems, 3) hyperactivity, and 4) peer relationship problems. There are five questions for each domain. Item 1–4 constitutes the mental health problem symptoms and can be aggregated into a single total difficulties score (TDS). The entire questionnaire took about 30 minutes. English and French are the administrative languages in Uganda and Burkina Faso, respectively, and they are taught in school. All the written material was in these two languages. The relevant local languages, *Lumasaaba* and *Diola*, respectively, were not systematically taught in school so a written version of it was hard to use for the data collectors who spoke the local languages. We translated the questionnaire material items immediately underneath the English and French items so we had a standardised content validity translation of all items. This was done in group work to ensure concept validity across different local dialects. Back-translation was also done.

School performance was assessed by an age appropriate school test that was made up by and corrected by two qualified teachers. It embraced all the expected fields: Literacy, Writing, English and Maths. The test was consistent with the national curriculum and the lay-out was similar to the usual school tests given in the study area. The test took about 20–30 minutes for the children and was done only in Uganda.

Height was measured to the nearest 0.1cm using a Seca stadiometer^®^ in the 263 series. The anthropometric index height-for-age z-scores were calculated using the WHO Child Growth Reference 2007 (www.who.int/childgrowth/en).

### Covariates

Covariates included parental (mother and father) age, education and occupation, cooking fuels, house ownership, drinking water sources, child’s age, sex and school attendance as well as the household socio-economic status. For the assessment of socio-economic status, a rank was constructed based on the multiple component analysis function in Stata. The modelling included assets such as mobile phones, radios, bikes, scooters and house construction characteristics (roofing materials, window, walls and doors). If the variable had no ‘variance’ (<10 owned it or >90% owned it), it was considered as not adding value to the model and was withdrawn. The rank was divided into quintiles and the distribution presented by arm. To address content validity the quintiles were tabulated against goods such as electricity, car, tap water, computer and mobile phones to ensure that those items were distributed in the top quintiles. This was the case for our final model that explained 89.7% of the first dimension.

### Assessment

The data collection was carried out by different types of data collectors. For example, the neuro-psychological tests were done by personnel holding at least a university degree in psychology or a similar subject. They were trained and supervised by a senior psychologist who is an expert in the field (PB, PH). MB and BG supervised the research team on neuropsychological testing. The psychometric tests were done in the mornings, and snacks and refreshment were provided during the day to the caregiver-child pair. The SDQ was incorporated into the larger questionnaire. Data collectors who had received training in data collection and who had gained experience in various research projects within the consortium, collected the data.

### Data management and statistical analyses

Data was analysed with Stata version 12.0 (StataCorp LP, TX, U.S.). Categorical variables were summarized with percentages while means, medians, standard deviations, ranges and interquartile ranges were used for continuous variables. The main exposure variable was to have been randomized to an EBF peer counselling cluster in the first six months of life. The main outcomes, all of them continuous, were child’s cognitive function, behavioural symptoms, school performance, and physical growth. Age adjusted z-scores for the cognitive assessment and emotion-behaviour-social symptoms were generated from the control group. We estimated the association between the exposure and each of the continuous outcomes using linear regression. Potential confounding variables that were imbalanced at baseline and were associated with the outcomes with a p-value <0.25 were adjusted for using multivariable linear regression models. The country datasets were pooled and all final regression analyses were adjusted for clustering.

While regression analyses were limited to participants with complete data on all covariates, there were covariates with missing data including height-for-age z-scores. Multiple imputations (20 imputations) of missing data using chained equations imputations and assuming that the data was missing at random resulted in linear regression findings that were comparable to those obtained when analysis was restricted to only those with complete data.

#### Role of the funding source

The study was funded by Grand Challenges Canada’s Saving Brains programme from 2012: Saving Brains in Uganda and Burkina Faso (PROMISE SB) project number: 0064–03. The funder of the study facilitated regular meetings where grantees could attend and discuss selection of tools, methods and results. The intention was that this could ease comparability on the effectiveness of the interventions that got funding for follow-up. A set of indicators were thus agreed upon and the outcome measures selected for the PROMISE SB study were done in order to harmonise the study with these requirements. However, the researchers were free to select the tools according to our skills and experience. The sponsor had no role in data analysis, interpretation and report writing.

### Ethics

In Uganda, ethical approval was obtained from the Makerere University School of Medicine Research and Ethics Committee (REC. Ref. 2012–177) on 5th November 2012; and from the Uganda National Council for Science and Technology (Ref. SS 3123) on 22nd April 2013. In Burkina Faso, the study was approved by the institutional review board of Centre Muraz on 4th April 2013 (Ref. 008-2013/CE-CM). All participants provided written informed consent.

The trial was duly registered on 20th June 2013 on Clinical Trials.gov (NCT01882335). In Uganda, recruitment started in earnest in May 2013 and in Burkina Faso in July 2013. That means that in Burkina Faso, the study started one month after registration on Clinical Trials.gov. However in Uganda, it started one month before registration on Clinical Trials.gov. The authors regret the delay in registration. The authors confirm that all ongoing and related trials for this intervention are registered.

## Results

### Study profile and baseline characteristics

Study population profile, Fig 1, and baseline characteristics of the studied population are presented in Tables 1 and 2. The intervention and control group were similar with regard to sex of child, sex of primary caretaker, marital status and other socio-economic variables. The majority of caretakers (83%) reported to live in a marital relationship and to own their house (92%). The population in the study areas were generally poor with the majority being peasant farmers (91% for mothers and 70% for fathers), used wood as main source of cooking energy (85%) and used surface water for drinking (67%). Socio-economy, electricity and duration of attendance in kindergarten were slightly in favour of the control-group, and these covariates were adjusted for in the final models. The participants not reached in PROMISE SB in Uganda and Burkina Faso were generally similar across arms with respect to gender and socio-economic parameters at inclusion (S1 and S2 Tables).

**Table 1:** Baseline characteristics, continuous variables given.

	Intervention		Control	
	N	mean (SD)	N	mean (SD)
Child’s age at SDQ^a^ interview (years)	521	7.4 (0.6)	530	7.4 (0.6)
Child’s age at psychometric testing (years)	521	7.1 (0.5)	532	7.1 (0.5)
Child’s age at school testing (years) ^b^	124	8.2 (0.5)	131	8.1 (0.6)
Mother’s age (years)	472	33.4 (6.5)	475	33.2 (6.5)
Father’s education (years)^b^	305	7.0 (3.2)	289	7.2 (3.6)
Mother’s education (years)^b^	291	6.0 (2.9)	275	6.2 (3.3)
Number of people in household	520	8.5 (3.9)	530	9.2 (5.0)
Number of bedrooms in home	518	2.5 (1.4)	529	2.7 (1.6)
Mother’s number of children	515	5.3 (2.4)	525	5.2 (2.5)
Time spent in kindergarten (months)^c^	158	16.5 (10.0)	144	19.1 (10.3)
Time spent in primary school (months)^c^	165	10.8 (7.6)	173	10.3 (6.9)

^a^SDQ = Strengths and difficulties questionnaire; ^b^Including Ugandan data only

^b^Adult education is recorded in completed academic school years so that primary 6 is equivalent to 6 years in school, etc.

^c^Childrens time in school is counted irrespectively of grade or level.

**Table 2:** Baseline characteristics, categorical variables given.

	Intervention	Control
	522	530
	N (%)	N (%)
Sex of child (male)	254 (48.7)	272 (51.3)
Primary caregiver		
Mother	233 (44.6)	221 (41.7)
Father	242 (46.4)	250 (47.2)
Other	47 (9.0)	59 (11.1)
Respondent is married	430 (82.7)	441 (83.4)
Polygamy (father has more than 1 wife)		
Yes	226 (43.3)	238 (44.9)
Father says he can read	273 (52.7)	266 (50.5)
Father’s Occupation		
Peasant	357 (68.4)	382(72.1)
Commercial farmer or shop keeper	133 (25.5)	100 (18.9)
Other	32 (6.1)	48 (9.1)
Mother says she can read	207 (40.0)	206 (39.1)
Mother’s Occupation		
Peasant	475 (91.4)	487 (92.1)
Commercial farmer or shop keeper	22 (4.2)	27 (5.1)
Other	23 (4.4)	15 (2.8)
Socioeconomic status quintile		
1 (poorest)	105 (20.1)	107 (20.2)
2	120 (23.0)	97 (18.3)
3	120 (23.0)	104 (19.6)
4	83 (15.9)	105 (19.8)
5 (least poor)	94 (18.0)	117 (22.1)
Electricity (yes)	262 (50.2)	311 (58.7)
Fuel for cooking		
Wood	440 (84.3)	455 (85.9)
Charcoal	76 (14.6)	69 (13.0)
Other	6 (1.1)	6 (1.1)
Drinking water		
Surface water	347 (66.5)	352 (66.4)
Borehole or tap	172 (32.9)	177 (33.4)
Other	3 (0.6)	1 (0.2)
House ownership		
Own	482 (92.3)	479 (90.4)
Rent	37 (7.1)	48 (9.0)
Other	3 (0.6)	3 (0.6)
School attendance for study child		
No	145 (27.8)	162 (30.6)
Ever attended kindergarten or primary^a^	276 (52.9)	271 (51.1)
Attended both kindergarten and primary^b^	95 (18.2)	91 (17.2)

^a^Kindergarten could be attended from 3–4 years and is not free

^b^School starts from the age of 6–7 in the two countries and is free, but many parents complain about costs related to transport, uniforms and equipment

### Outcomes

#### Cognitive abilities

Looking at the pooled estimates for Uganda and Burkina Faso, no statistically significant difference was seen for any of the aggregated or single subscales at the K-ABC2 including general cognition, and working memory. This was the same for the TOVA test including attention and inhibition and for cognitive flexibility using CCT in both the unadjusted and adjusted analysis (Table 3).

**Table 3:** Neuro-psychological testing results from KABAC II (General cognition, MPI and Working memory), T.O.V.A (attention and inhibition) and CCT (cognitive flexibility). Country-pooled results showing linear regression unadjusted and adjusted models with trial arm as the dependent variable.

Domain	Unadjusted mean difference^a^ (95% CI)	Adjusted mean^b^ difference (95% CI)
General cognition, MPI, N = 1028	0.08 (-0.13 to 0.29)	-0.07 (-0.30 to 0.15)
Working memory, N = 1027	-0.01 (-0.32 to 0.29)	-0.07 (-0.29 to 0.16)
Attention, N = 1014	0.12 (-0.02 to 0.26)	0.11 (-0.13 to 0.35)
Inhibition, N = 1014	0.03 (-0.17 to 0.23)	-0.05 (-0.28 to 0.19)
Cognitive Flexibility, N = 1026	-0.07 (-0.21 to 0.07)	0.02 (-0.27 to 0.30)

^a^Adjusted for the design effect (clusters) only

^b^Adjusted for socioeconomic status, electricity in home, duration in kindergarten and cluster

#### Emotional development

No statistically significant difference was seen for any of the behavioural symptoms scales or the aggregated symptom score (Table 4).

**Table 4:** Emotional-behavioural symptom results from the Strengths and Difficulties questionnaire, parent version. Country-pooled results showing linear regression unadjusted and adjusted models with trial arm as the dependent variable, N = 1048.

Symptom area	Unadjusted mean difference^a^ (95% CI)	Adjusted mean^b^ difference (95% CI)
1. Emotional symptoms	0.11 (-0.07 to 0.29)	0.11 (-0.11 to 0.33)
2. Conduct problems	0.05 (-0.12 to 0.22)	0.04 (-0.18 to 0.27)
3. Hyperactivity symptoms	-0.02 (-0.19 to 0.14)	-0.05 (-0.26 to 0.17)
4. Peer relationship problems	-0.09 (-0.33 to 0.15)	0.12 (-0.09 to 0.34)
Total problems, summing 1–4	0.02 (-0.18 to 0.22)	0.08 (-0.12 to 0.27)

^a^Adjusted for the design effect (clusters) only

^b^Adjusted for socioeconomic status, electricity in home, duration in kindergarten and cluster

#### School performance and growth

The children also scored similarly on school tests in the pooled estimates and were equally high for their age (Table 5), however, in both arms their mean height-for-age z-scores were around -1 standard deviation from the mean.

**Table 5:** Average school test results and height for age z-scores (HAZ) by intervention and control arms.

	Intervention		Control	
	N	mean (SD)	N	mean (SD)
School grades (total:100credits, available for Uganda only)	122	79.7 (21.2)	131	80.0 (22.1)
Height for age z-scores (both countries)	493	-1.0 (1.0)	523	-1.0 (1.1)

#### Subgroup analysis

Subgroup analysis of the neuro-psychological outcomes as well as the height for age z scores did not yield any statistically significant differences between the EBF and non-EBF children, except for inhibition (adjusted mean difference 95% CI: 0.24 (0.02 to 0.46) (Tables 6 and 7).

**Table 6:** Neuro-psychological testing results from KABAC II (General cognition, MPI and Working memory), T.O.V.A (attention and inhibition) and CCT (cognitive flexibility). Country-pooled results showing linear regression unadjusted and adjusted models with EBF status at 12 weeks as the dependent variable, 1-week recall.

Domain	Unadjusted mean difference^a^ (95% CI)	Adjusted mean difference^b^ (95% CI)	ICC, cluster
General cognition, MPI	0.07 (-0.11 to 0.24)	0.14 (-0.12 to 0.41)	0.12
Working memory	0.04 (-0.15 to 0.23)	0.07 (-0.19 to 0.31)	0.06
Attention	0 (-0.13 to 0.13)	0.06 (-0.17 to 0.29)	0.02
Inhibition	0.03 (-0.15 to 0.20)	*0*.*24 (0*.*02 to 0*.*46)*	0.05
Cognitive Flexibility	-0.02 (-0.15 to 0.09)	0.05 (-0.29 to 0.19)	0.01

^a^Adjusted for the design effect (clusters) only

^b^Adjusted for socioeconomic status, electricity in home, duration in kindergarten and cluster

**Table 7:** Emotional-behavioural symptom results from the Strengths and Difficulties questionnaire, parent version. HAZ: Height for age z-score. Country-pooled results showing linear regression unadjusted and adjusted models with EBF status at 12 weeks as the dependent variable, 1-week recall.

Symptom area	Unadjusted mean^a^ difference (95% CI)	Adjusted mean^b^ difference (95% CI)	ICC^c^, cluster
1. Emotional symptoms	0.06 (-0.21 to 0.09)	-0.05 (-0.31 to 0.21)	0.05
2. Conduct problems	-0.08 (-0.23 to 0.07)	-0.07 (-0.31 to 0.16)	0.03
3. Hyperactivity symptoms	0.01 (-0.14 to 0.17)	0.03 (-0.31 to 0.39)	0.01
4. Peer relationship problems	0.09 (-0.03 to 0.23)	-0.04 (-0.28 to 0.20)	0.11
Total problems, summing 1–4	-0.01 (-0.17 to 0.15)	-0.04 (-0.31 to 0.22)	0.08
HAZ	0.02 (-0.19 to 0.24)	-0.04 (-0.43 to 0.35)	0.10

^a^Adjusted for the design effect (clusters) only

^b^Adjusted for socioeconomic status, electricity in home, duration in kindergarten and cluster

^c^Intra-cluster coefficient of variation

## Discussion

The present study assessed the long-term effect of an exclusive breastfeeding (EBF) promotion intervention on child cognitive abilities, social emotional development, school performance and linear growth in pooled estimates from two sub-Saharan low-income countries, Uganda and Burkina Faso. As none of the neuro-psychological tests and questionnaire based instruments were normed in sub-Saharan Africa, all scores were converted into age specific age bands of 1 year z-scores that were compared between arms.

Our main findings suggest no association between EBF promotion and the studied outcomes. Separate country assessment did not show any relationship, and pooling the data did not “wash out” a minor effect in any site. As the large sample size made this a study with high power we think ‘type II’ error (not rejecting H0 when H0 is not true) is less likely. We provided post-hoc calculations in an earlier paper on secondary outcomes [19], however, this is not a recommended practice [32] and is strongly discouraged in the literature. Looking at the confidence intervals, they all narrow and contain zero. Looking at national, pooled, sub- and aggregated scales increases our confidence that there were no differences detected in cognitive abilities and social-emotional development from the peer support breastfeeding promotion on those mental health aspects at 5–8 years. We provided intra-cluster correlation coefficients for future sample size calculations (Tables 6 and 7) when stratifying on EBF-practice at 12 weeks. We also re-ran our models stratifying for sex. There wereno sex difference on the cognitive performance tests or social-emotional development in the pooled data. Both children in the intervention and control arms were equally distributed in terms of socio-demographic characteristics, except with a socio-economic favour of the control arm. Controlling for that did not change our results. Further, the children re-enrolled shared similar characteristics to the children lost to follow-up, so we did not expect a major selection bias. Our findings are contrary to findings from other countries where EBF promotion has been associated with child cognitive development [10].

To the best of our knowledge, EBF promotion has not yet been identified as an intervention that can improve cognitive performances among children in sub-Saharan Africa. Even though we did not find an association between EBF promotion and children’s test performance, social emotional development and linear growth outcomes in the present study, there are possible explanations that might be related to the characteristics of the intervention and the control arm in the studied cohort.

First, the majority of our children in the cohort were breastfed, as breastfeeding is the norm [33]. It may be harder to detect an effect where the frequency of EBF varies in a population predominantly breastfed compared to studies comparing breastfeeding to non-breastfeeding practices [3]. Thus, the beneficial effect of the intervention could have been diluted. This problem of comparing the best (exclusively breastfeeding) to the second best option (any breastfeeding) was also mentioned in the PROBIT trial follow-up published in 2008, where exclusive breastfeeding promotion of varying time durations were compared [10]. However, they managed to detect favourable outcomes related to the intervention. If we only look at the outcome measures by the primary outcome of the trial, exclusive breastfeeding at three months using a one week recall, we still do not find any difference in cognition, except some improvement in the adjusted analysis for inhibition (Table 6). This was not present when we looked at the six months EBF status. As the inhibition was the only significant different finding, we interpret that with care. Similarly, a recent cohort study from South Africa among HIV exposed and unexposed children [34] only found improved cognition among boys who had been breastfed for more than 5 months compared to less than one months, indicating that in order to detect a difference, the feeding behaviour needs to be substantially different.

Factors such as nutrition, home environment, parental education and family income have been identified as contributing to improve cognitive abilities among children in sub-Saharan Africa [27, 35, 36]. Domestic violence, harmful substance use, extreme poverty, sickness or undernutrition do affect children performances negatively [37]. Children in the cohort may have been exposed to some of these conditions and challenges that may have wiped out any smaller detectable benefit from the intervention.

Particularly in sub-Saharan Africa, breastfeeding promotion and large Unicef/WHO initiatives such as the baby-friendly-hospital initiative were challenged by the HIV-epidemic since mother-to-child transmission of breastfeeding was described in the early nineties [38]. The frequent shifts in the WHO recommendations on HIV and infant feeding, after the millennium, was appreciated for its scientific progress, but often created confusion and stress [39]. In 2006, when the PROMISE EBF trial started, the 2004 WHO HIV and infant guideline [40] emphasized the so called “AFASS” criteria (acceptable, feasible, affordable, sustainable and safe) for replacement feeding to be recommended. In case of any HIV infection, the likelihood for the woman to be tested and included in a prevention-of-mother-to-child program was limited [41]. The PROMISE EBF research team did not have the capacity to include HIV assessment or management as a component in the initial trial. That we were not be able to stratify our results by HIV-exposure status is a major limitation. A strength however was the one-week recall period of Exclusive Breastfeeding and concurrent follow-up. This makes us confident in describing the feeding practice.

It may also be that the intervention was not intensive or long enough. We know that the intervention was pro-poor [20] attracting the poorest women most towards adapting it. These women might thus have prolonged EBF into the recommended period of complementary feeding which our intervention did not cover.

Even if we attempted, through stringent supervision and training in psychometric testing, to get as good data as possible one has to remember that none of the tests were normed in Uganda or Burkina Faso. However, they have been successfully used and reported with good construct validity in western and eastern Africa, including Uganda [29]. We believe that the conversion into z-scores, which were then compared between the two arms, would have allowed a detection of any substantial arm differences. We did not control for parental or caregivers cognitive performance or mental health status either, however, we believed that this was handled with randomisation.

Moreover, child cognition and behaviour are not static, but are part of a developmental process. As we cannot control for all time dependent variables, we cannot conclude with confidence that there were no beneficial effects of the EBF promotion intervention except behavioural change [12]. Mothers reported satisfaction with the intervention [16] which also matters. Rather it might be correct to say that we were not able to notice differences between EBF promotion and control arms for tested outcomes after 5–8 years’ follow-up.

Further, our results suggest no association between EBF promotion and children schooling outcomes in the two arms, although children in the control arm were better at attending kindergarten. It is hard to interpret how this can be related to the intervention, and there is a need to consider other factors in place for more accurate interpretation, such as capturing of socio-economic status.

No behavioural problems were associated with the EBF promotion intervention. In Burkina Faso and Uganda, no advantageous effects of EBF promotion were noticed on child growth at six months, rather a small negative development [19] even after adjusting for socio-economic status. The current study did not find any difference in HAZ between the intervention and control arms. This suggest a temporal fluctuation and the absence of any harm from the intervention.

This study had some limitations related to our study population. The main limitation is related to the selected tools, which were not normed in the settings, may not have given a correct picture of the children’s ability to perform or were not culturally sensitive to their behavioural symptoms. All children have lots of capabilities and capacities we are not measuring like humour, self-esteem, belief-systems, risk-taking and trust. There is a need for improved culturally-sensitive contextually relevant and normed instruments which can be used particularly for evaluating interventions and detecting treatment gaps in children.

## Conclusions

Peer promotion for exclusive breastfeeding in Burkina Faso and Uganda was not associated with differences at 5–8 years of age in a range of measures of child development: cognitive abilities, emotion-behaviour-social symptoms or linear growth. This study from sub Saharan Africa did not reconfirm findings elsewhere that have shown an association between exclusive breastfeeding and cognitive performance. This might be due to a number of methodological limitations inherent in the current study. For example since the majority of the children were breastfed, the benefits of the intervention could have been diluted. Other factors such as the mental and HIV status of the mothers (which were not assessed in the current study) could have affected our results. Hence regarding the effect of exclusive breastfeeding on measures of child neurocognitive development in sub Saharan Africa, the jury is still out.

## Supporting information

S1 Table Baseline characteristics of the children studied in PROMISE Saving Brains and those not re-enrolled. Continuous variables.
(DOCX)

S2 Table Baseline characteristics of the children studied in PROMISE Saving Brains and those not re-enrolled. Categorical variables.
(DOCX)

S3 Table Post hoc power assessment. (DOCX)

S1 File CONSORT checklist: USE_CONSORT Checklist_August_2017.doc. (DOC)

S2 File Protocol for the Saving Brains study in Burkina Faso and Uganda: USE_PROTOCOL_Saving Brain_BFA_UGA.doc. (DOC)
