# Supplementary material for: Exclusive breastfeeding promotion and neuropsychological outcomes in 5-8 year old children from Uganda and Burkina Faso: Results from the PROMISE EBF cluster randomized trial
Source: PLoS One. 2018 Feb 23;13(2):e0191001. doi: 10.1371/journal.pone.0191001 (PMC5824999; doi:10.1371/journal.pone.0191001)
Supplement: S1 Table — Continuous variables. (DOCX) [file pone.0191001.s001.docx]

**S1 Table: Baseline characteristics of the children studied in PROMISE Saving Brains and those not re-enrolled**

Continuous variables

| Baseline information | Participation in Promise Saving Brains | |  | Not re-enrolled in Promise Saving Brains | |
| --- | --- | --- | --- | --- | --- |
|  | N | mean (95%CI) |  | N | mean (SD) |
| Mother’s age | 954 | 26.2 (6.5) |  | 409 | 24.6 (6.5) |
| Mother’s education | 1049 | 3.4 (3.8) |  | 461 | 3.7 (4.1) |
| Mother’s BMI | 785 | 21.9 (2.7) |  | 331 | 21.6 (2.3) |
| Monthly income (Euro 2011) | 626 | 10.9 (25.7) |  | 259 | 9.9 (17.6) |
