# Supplementary material for: Exclusive breastfeeding promotion and neuropsychological outcomes in 5-8 year old children from Uganda and Burkina Faso: Results from the PROMISE EBF cluster randomized trial
Source: PLoS One. 2018 Feb 23;13(2):e0191001. doi: 10.1371/journal.pone.0191001 (PMC5824999; doi:10.1371/journal.pone.0191001)
Supplement: S2 Table — Categorical variables. (DOCX) [file pone.0191001.s002.docx]

**S2 Table: Baseline characteristics of the children studied in PROMISE Saving Brains and those not re-enrolled**

Continuous variables

| Socioeconomic quintile group at baseline | Participation in Promise Saving Brains | Non-participation in Promise Saving Brains |
| --- | --- | --- |
| Lowest | 228 (21.0) | 88 (18.5) |
| 2 | 238 (21.9) | 99 (20.8)) |
| 3 | 210 (19.4) | 76 (16.0) |
| 4 | 214 (19.7) | 102 (21.5) |
| Highest | 194 (17.9) | 109 (22.9) |
