# Supplementary material for: Exclusive breastfeeding promotion and neuropsychological outcomes in 5-8 year old children from Uganda and Burkina Faso: Results from the PROMISE EBF cluster randomized trial
Source: PLoS One. 2018 Feb 23;13(2):e0191001. doi: 10.1371/journal.pone.0191001 (PMC5824999; doi:10.1371/journal.pone.0191001)
Supplement: S3 Table — (DOCX) [file pone.0191001.s003.docx]

**S3 Table: Post hoc power assessment**

The power reflect power to the “actual difference” not the any clinically assumed relevant difference (e.g. SD=0.2)

| Domain | Unadjusted mean difference^a^ (95% CI) | Adjusted mean^b^ difference (95% CI) | Power^a^ |
| --- | --- | --- | --- |
| General cognition, MPI, N=1028 | 0.08 (-0.13 to 0.29) | -0.07 (-0.30 to 0.15) | 0.025 |
| Working memory, N=1027 | -0.01 (-0.32 to 0.29) | -0.07 (-0.29 to 0.16) | 0.05 |
| Attention, N=1014 | 0.12 (-0.02 to 0.26) | 0.11 (-0.13 to 0.35) | 0.52 |
| Inhibition, N=1014 | 0.03 (-0.17 to 0.23) | -0.05 (-0.28 to 0.19) | 0.07 |
| Cognitive Flexibility, N=1026 | -0.07 (-0.21 to 0.07) | 0.02 (-0.27 to 0.30) | 0.07 |
| Emotional (SDQ) N=1048 | 0.11 (-0.07 to 0.29) | 0.11 (-0.11 to 0.33) | 0.43 |

^a^ Post hoc power calculation was done in Stata using ‘sampsi’ and the following formulae:

sampsi mean1 mean2, n1(#) n2(#) sd1(#) sd2(#) a(0.05) where mean refer to the mean value in the two arms (1+2), the given standard deviations of the two arms and the given population in the two arms. Post hoc power calculation is not recommended given the nature of the calculations: the smaller the difference, the ‘less’ power we have.
