# Supplementary material for: Exclusive breastfeeding promotion and neuropsychological outcomes in 5-8 year old children from Uganda and Burkina Faso: Results from the PROMISE EBF cluster randomized trial
Source: PLoS One. 2018 Feb 23;13(2):e0191001. doi: 10.1371/journal.pone.0191001 (PMC5824999; doi:10.1371/journal.pone.0191001)
Supplement: S2 File — (DOC) [file pone.0191001.s005.doc]

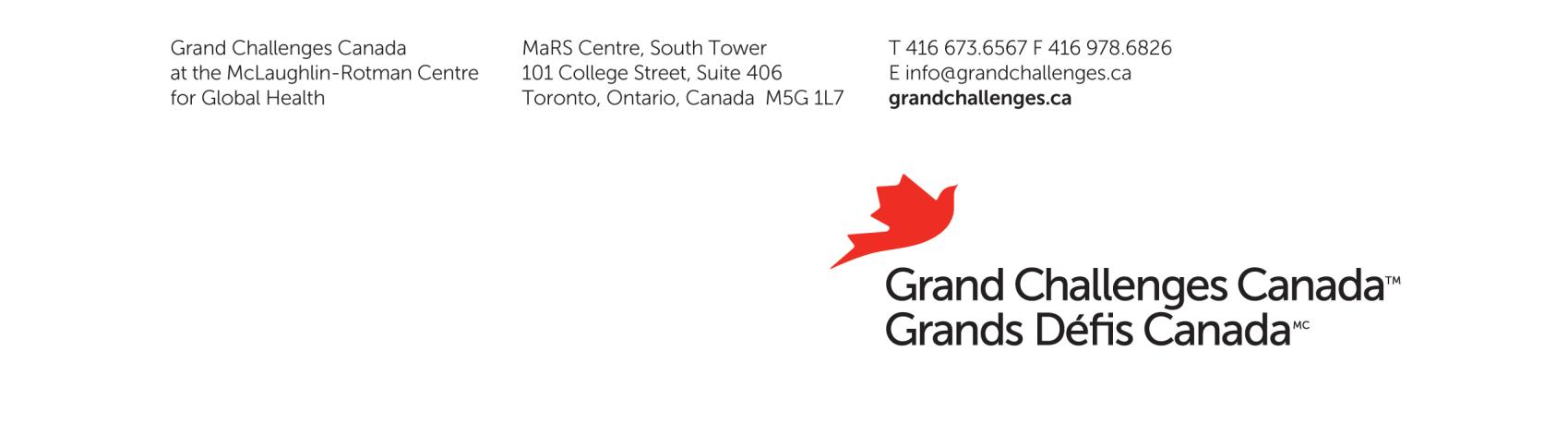


**SAVING BRAINS IN UGANDA AND BURKINA FASO (PROMISE-SB)**

**A RESEARCH PROPOSAL SUBMITTED TO SCHOOL OF MEDICINE RESEARCH AND ETHICS COMMITTEE**

**MAKERERE UNIVERSITY COLLEGE OF HEALTH SCIENCES**

**PROTOCOL VERSION 1.1**

**AUGUST 2012**

**Signatures**

I, the Principal Investigator, agree to conduct the study in full accordance with the provisions of this protocol and will comply with all requirements regarding the obligations of clinical investigators as outlined in the principles of Good Clinical Practices (GCP) which I have read. I agree to maintain all study documents until the study sponsor consents to disposal of files in writing.

I will ensure that all associates, colleagues, and employees assisting in the conduct of the study are informed about the obligations incurred in their contribution to the study.

**Principal Investigator** Date

James K Tumwine

Makerere University College of Health Sciences, Uganda

**Co-Principal Investigator** Date

Nicolas Meda

Centre Muraz, Burkina Faso

The protocol development was a collaborative initiative of the PROMISE SB study group

**Sponsorship**

**Sponsor**: Grand Challenges Canada (Grant number: #0064-03)

**In kind contribution**: Makerere University College of Health Sciences (Uganda), Centre Muraz

(Burkina Faso), University of Bergen (Norway)

**Co-investigators on the study**

Victoria Nankabirwa Makerere University Co-Investigator

Hama Diallo: Centre Muraz Co-Investigator

Grace Ndeezi: Makerere University Co-Investigator

Angelina Kakooza: Makerere University Co-Investigator

Thorkild Tylleskär: University of Bergen Co-investigator

Ingunn Engebresten: University of Bergen Co-Investigator

Irene B Elgen: University of Bergen Co-Investigator

**TABLE OF CONTENTS**

list of abbreviations and acronyms ............................................................................................... 5

Executive Summary ..................................................................................................................... 7

1.0 PROJECT PURPOSE AND BACKGROUND ................................................................... 9

1.1 LITERATURE REVIEW..................................................................................................... 9

2.0 PROBLEM STATEMENT ................................................................................................ 14

2.1 STUDY JUSTIFICATION................................................................................................. 15

2.2 AIMS AND OBJECTIVES ................................................................................................ 15

Primary objective ............................................................................................................15

Secondary objective .......................................................................................................15

3.0 STUDY PROCEDURES ................................................................................................... 16

4.0 Data collection and management ....................................................................................... 24

Statistical analysis ..........................................................................................................25

5.0 Ethical Considerations ....................................................................................................... 25

5.1 Institutional/Organizational and Management Capacity.................................................... 26

Appendix I Project Framework Table ....................................................................................... 29

Citations...................................................................................................................................... 35

APPENDIX II: INFORMED CONSENT FORM ...................................................................... 38

**LIST OF ABBREVIATIONS AND ACRONYMS**

BMI Body Mass Index

BP Blood Pressure

CRP C- Reactive Protein

EBF Exclusive Breast Feeding GPS Global Positioning System IQ Intelligence Quotient

MRI Magnetic Resonance Imaging MUAC Mid- Upper Arm Circumference NGO Non-governmental Organization WHO World Health Organisation

**Operational Definitions**

**Exclusive breastfeeding**: Feeding only breast milk (including expressed breast milk) and allowing the baby to receive vitamins, minerals or medicine**.**

**Human Capital Formation:** The process by which a given individual achieves their highest potential and aspirations by integrating and optimizing a combination of ongoing processes such as education, job seeking, employment, skill formation, and personal development.

**Executive Summary**

**Background**

There is some evidence to show that exclusive breastfeeding (EBF) improves cognition and other

determinants of human capital formation. Most of this evidence has come from outside of sub Saharan Africa where EBF is on the decline for various reasons such as the HIV pandemic and aggressive marketing of breast milk substitutes. Promotion of EBF in sub Saharan Africa has hitherto depended on health systems which are struggling with serious shortages of human resources and underfunding. Between 2006 and 2008 we carried out a cluster randomized study (PROMISE-EBF) to establish the effect of individual home-based peer counseling on EBF. The results (recently published in the *Lancet*) were remarkable. This cluster randomized trial which compared mothers in the intervention arm that were offered at least 5 peer counseling visits promoting exclusive breastfeeding (EBF) to mothers in the control arm, that received the standard of care showed a doubling in the prevalence of EBF in the intervention arm. Community based peer counselors increased exclusive breastfeeding in Uganda and Burkina Faso from about 40% to 80%. The peer counselors were paid about 10 US dollars per month. Given these findings, we are curious to find out the effect of peer counseling for EBF on human capital formation in an African setting.

**Purpose**

Using an integrated innovative approach, this study will tackle scientific, business and social

barriers to the use of peer counselling for EBF on human capital formation in Burkina Faso and Uganda. From the scientific angle, the PROMISE-SB study will provide the first set of data from a randomized trial in an African context on peer counselling for EBF on human capital formation. It will contribute to a crucial wider discussion on external validity regarding the relationship between exclusive breastfeeding and intellectual performance and mental health. In the social area, the PROMISE-SB study will identify solutions to potential barriers that limit scale up for peer counselling for EBF. From the business perspective, the study will identify the best business model for affordable, acceptable and cost effective ways of delivering peer counselling for EBF in Burkina Faso and Uganda. If this intervention is found effective, and is adopted, it could improve productivity in adulthood and work towards breaking the cycle of poverty.

**Approach**

We anticipate locating and re-enrolling a sizable proportion of the PROMISE-EBF cohort in

Burkina Faso and Uganda. Though the scheduled follow-up of children in the EBF-trial was up to

6 months, follow-up studies have been carried out and published from Burkina Faso at 12 months of age and in Uganda at 2 years of age. These follow up studies had very good overall follow up rates of over 70%, just like the original study, because of the involvement of members in the local communities. We used recruiters from the clusters to identify the pregnant women. The same recruiters were involved in the follow-up studies in Burkina Faso and Uganda. We also have access to Global Positioning System (GPS) data for the homesteads which, in these rural communities, have no street numbers. We estimate that within a period of 3 months our teams will be able to retrieve over 70% of children initially enrolled and who are still alive. We will

request for re-consent for inclusion in the PROMISE-SB study. Data collection is expected to last

12 months.

**Organizational and investigator capacity**

Our teams in Uganda and Burkina Faso are led by experienced scientists who are trained in

epidemiology, mother and child health, psychology and psychiatry, with experience in conducting community-based randomized controlled trials. We also have well trained teams of research assistants who are based in the communities. They will be an asset in retrieving the PROMISE- EBF children. We have particularly very good relationship with the communities and regularly meet members of the community through Community Advisory Groups. Both teams work closely with the respective ministries of health. We look forward to this very promising study.

**Project Summary**

The PROMISE Saving Brains (PROMISE-SB) study will include children born in the cohorts

established in Uganda and Burkina Faso between 2006 and 2008 for the PROMISE- EBF trial [1]. Using these cohorts, the PROMISE-SB study will give us a unique opportunity to assess whether EBF promotion enhances human capital formation and to assess mental health and cognitive function among 5-7 year old children in these two countries.

**1.0 PROJECT PURPOSE AND BACKGROUND**

Many challenges occurring in the prenatal period or in the first 1000 days of life put children at risk for brain disorders, often termed developmental disabilities because they affect children, at a particularly vulnerable stage, slowing or halting the development of the nervous system [2]. Poverty and inadequate access to health care and education, infection and trauma, as well as caregiver mental health problems affect large proportions of the children in Uganda and Burkina Faso. Although not well studied in sub-Saharan Africa, nutrition, particularly breastfeeding has been recognized in other settings as an important determinant of cognitive functioning [3].

Using an integrated innovative approach, this study will tackle scientific, business and social barriers to the use of peer counselling for EBF on human capital formation in Burkina Faso and Uganda.

From the scientific angle, the PROMISE-SB study will provide the first set of data from a randomized trial in an African context on peer counselling for EBF on human capital formation. It will contribute to a crucial wider discussion regarding the relationship between exclusive breastfeeding and intellectual performance and provide useful descriptive information on mental health called for by WHO [4]. In the social area, the PROMISE-SB study will identify solutions to potential barriers that limit scale up for peer counselling for EBF in the first six months of life. From the business perspective, the study will identify the best business model for affordable, acceptable and cost effective ways of delivering peer counselling for EBF in Burkina Faso and Uganda. If this intervention is found effective, and is adopted, it could improve productivity in adulthood and work towards breaking the cycle of poverty.

**1.1 LITERATURE REVIEW**

*Breastfeeding and cognitive function: the epidemiology*

A recent review on breastfeeding and cognitive function concluded that there is a likely positive relation between breastfeeding and increased cognitive function [5]. Despite this conclusion, challenges still exist with regard to research on early child feeding and cognitive function. For example, most studies in which a positive association between breastfeeding and cognitive function has been found are from high-income countries. Also, among those studied, the improved breastfeeding practices including both longer duration of breastfeeding and higher degree of exclusive breastfeeding were found among women with higher social status and education. Moreover, many studies have been observational in design and even though attempts were made to control for confounding, residual confounding and reverse causation could explain part of the observed association [3]. It is also still unclear if it is a constituent of breast milk such as long-chain polyunsaturated fatty acids (LCPFA) or whether it is the act of breastfeeding associated with stimulation that enhances intellectual performance [5].

Existing reviews are to a large extent based on research from high-income countries and may not be representative of a wider global context. A few Asian studies diverged regarding the effect of

breastfeeding on cognition [6, 7]. A quick PubMed search entering ‘breastfeeding and cognition’ yielded around 200 hits. Adding ‘Africa’ reduced the hits to less than a handful of papers, none of which were relevant to this topic. A recent study by Brion and colleagues compared the results on breastfeeding and cognition from high and low income cohorts [3]. They included the British Avon Longitudinal Study of Parents and Children (ALSPAC) (N=5000) and the Brazilian Pelotas cohorts (N=1000). In addition they included a couple of randomized trials of breastfeeding promotion from low- and middle-income countries. They studied blood pressure (BP), body mass index (BMI) and intelligence quotient (IQ). In the British cohort breastfeeding was associated with lower BP, BMI and higher IQ after adjusting for confounders. This was not seen in the Pelotas cohort: BP and BMI were not associated with breastfeeding, while breastfeeding was associated with higher IQ. The authors concluded that breastfeeding could have a causal effect on IQ at the population level and suggested that any residual confounding in the association between breastfeeding and IQ could be overcome by comparing studies from different populations. The PROMISE SB study could, to a large extent, increase the external validity of the study question by embracing low-income countries (LIC) in Africa. In addition, excellent measurement of the breastfeeding intervention in the original PROMISE-EBF study will help the PROMISE-SB study to overcome some of the methodological challenges experienced by previous studies.

*Age*

Even with a good study design focusing on the relationship between breastfeeding and cognitive function it is worth noting that a variety of conditions can affect intelligence after the breastfeeding period. In other words, if breastfeeding is a protective factor for improved human capital embracing intellectual performance, factors such as trauma, sickness and sub-optimal psycho-social conditions can dilute the observed effects. It is therefore essential to start assessing cognitive function early enough before other influences have a major effect and late enough to get valid results of cognitive function [8]. We assume that a first assessment in middle childhood (5-7 years) will not only yield valuable data before many other life events influence cognitive function, but will also provide baseline data for future assessments.

*Existing knowledge on the relationship between breastfeeding and cognition/intellectual performance*

Anderson and colleagues published the first meta-analysis including 20 studies assessing the effect of breastfeeding compared to formula feeding on cognition [9]. Benefits in cognitive function were seen both in the unadjusted (5.32 (95% CI 4.51-6.14) IQ-points) and the adjusted analysis (3.16 (95% CI 2.35-3.98) IQ-points). A later review was done by a WHO-working group published in 2007 including 9 studies (some of them overlapping with Anderson) [4]. They reconfirmed an approximate mean difference of 5 IQ-points in favour of breastfed infants compared to non-breastfed infants (figure 1).

The Anderson *et al* review also showed a time-dependent association, with a longer breastfeeding duration being associated with an increasing difference in cognitive points between breastfed and formula fed groups. Michaelsen and colleagues also reported a similar temporal relation [5]. They showed that an increased duration of breastfeeding reduced the proportion of children with IQ <

90 points. Furthermore, according to Anderson *et al*, infants with low birth-weight benefitted

more from breastfeeding than normal weight infants with respect to cognitive development score

(table 1).

Figure 1: Mean difference in cognitive development scores and its 95% confidence interval

between breastfed and non-breastfed subjects in different studies. males (M), females (F) and all (A) is indicated in parenthesis.

Whether the estimate was for

-fed and

*Anderson JW, Johnstone BM, Remley DT (1999) Breast-feeding and cognitive development: a meta-analysis. Am J Clin Nutr 70: 525-535.*

Table 1: Weighted mean difference in cognitive development score between breast formula-fed children from the meta-analysis by *Anderson et al*

With regard to human capital, Michaelsen and colleagues [3] also estimated the expected benefit on human capital using a model calculating special schooling expenditure saved with increased IQ. They estimated that the cost of special education in the US could be reduced from $4.5 billion

to $3.9 billion if the percentage of children that were predominantly breast-fed at four months was increased from 20% to 50%[10]. However, that same study highlighted the necessity of improved infant feeding measurement and categorisation in these estimates. Using rigorously collected infant feeding data collected in the original Promise-EBF trial, the Promise SB study will be able to adequately address this need.

Most studies have addressed breastfeeding and cognitive function by considering breastfed groups versus formula fed groups. However, we will assess child populations that have been largely breastfed and where the intervention has been to promote EBF; resulting in a doubling of the EBF rates in the intervention arm compared to the control arm. Thus, we are comparing two arms where most children are breastfed, but where almost 80% are exclusively breastfed around 3 months in the intervention arm.

Data on cognitive function and mental health in relation to EBF promotion has also been described from the well-known Canada-led PROBIT-study (Promotion of Breastfeeding Intervention in Belarus 1996-97 included more than 17000 babies with birth weight more than

2500g in a trial design) [11]. Results from this study showed the frequency of EBF practices at

43% and 6% in the intervention and the control group, respectively. Newly published evidence from this trial found no significant difference in strength and difficulty questionnaire (SDQ) results between the arms, but differences on subtests in the Wechlser Abbreviated Scales of Intelligence (WASI) inventory: children in the intervention group scored higher on vocabulary and similarities subtests than the control group. Moreover, the teachers also rated their reading and writing skills to be higher (tables 2 and 3).

Yet another study of the effect of breastfeeding on cognitive development showed an association between improved fine motor skills and breastfeeding duration [12] providing a neurobiological hypothesis of the observed association. [13]. Thus, there is a need to assess whether the reported findings will be confirmed in our settings through the PROMISE-SB trial.

*Kramer MS (2010) "Breast is best": The evidence. Early Hum Dev 86: 729-732.*

Table 2: PROBIT results: mean WASI scores


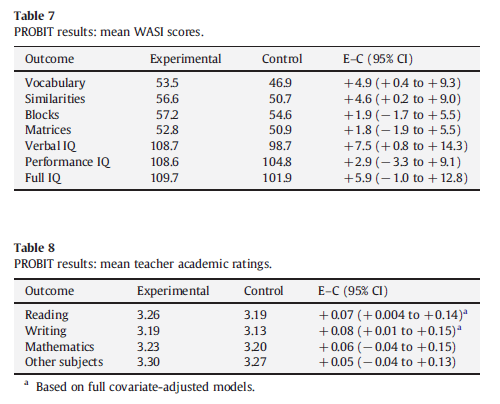


Table 3: PROBIT results: mean teacher academic ratings

*Kramer MS (2010) "Breast is best": The evidence. Early Hum Dev 86: 729-732. Theoretical framework*

Breastfeeding may contribute to cognitive development through either some constituent of breast milk or through the physical and social interactions associated with breastfeeding or a combination [13]. Human milk has higher concentrations of essential long-chain polyunsaturated fatty acids (LCPUFA) [13] and growth factors such as insulin-like growth factor (IGF) [14] than non-human milk and this could contribute to the observed cognitive differences. It is also evident from psychological research and clinical medicine that the mother’s behaviour affects the offspring’s mental capacity. Theories on attachment, secure and unsecure children, first described by Bowlby and Ainsworth decades back seem highly relevant in explaining a nutritive environment for cognitive development [14]. It is hypothesized that the increased frequency and duration of maternal-infant contact during breastfeeding could increase sensory and verbal interaction between mothers and their infants, and this could have a stimulatory effect on cognitive development [13]. In addition, studies on long-term epigenetic behavioural effects of licking and grooming by mother rats of their pups indicate that the physical and/or emotional act of breastfeeding could lead to permanent physiologic changes that accelerate neurocognitive development [14]. Based on these observations, it is plausible that breastfeeding enhances cognitive development and function. Michaelsen’s theoretical framework is in line with this thinking (figure 2) [5].

Figure 2: Conceptual framework showing how the behavioural pattern of the mother can be either a confounder or a mediator


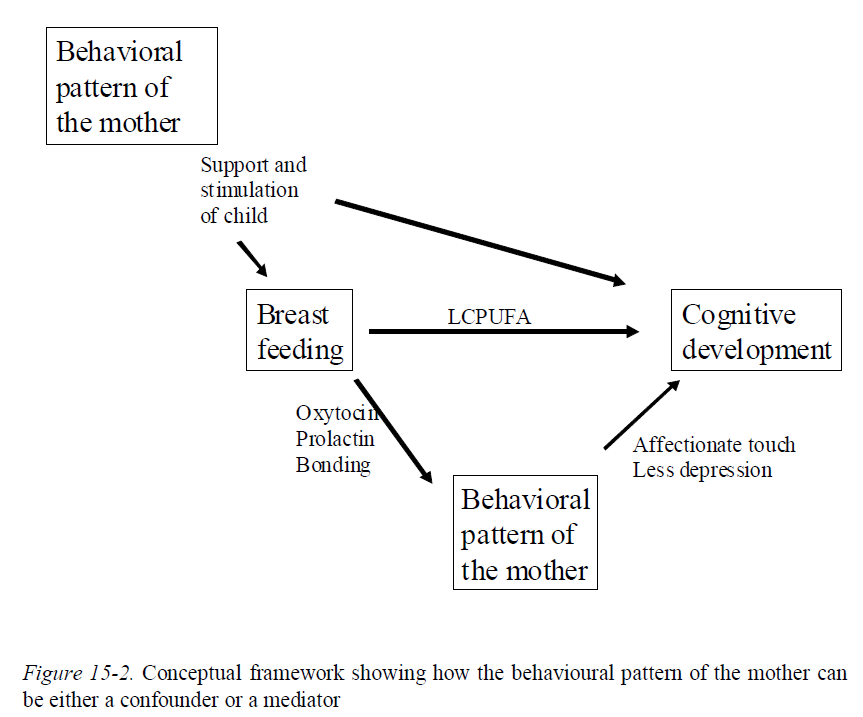


**2.0 PROBLEM STATEMENT**

*Need for global knowledge*

A recent paper from the Global Mental Health Lancet series 2 [15] stressed the evidence gaps in terms of prevalence, risk and protective factors and interventions to prevent and treat childhood and adolescent mental health problems. Non-specific interventions targeting early childhood including improved nutrition, early stimulation and improvement of carer sensitivity and responsiveness are mentioned as promising [15,16] In the web-appendix to the Lancet paper a few African studies were found from Ethiopia, South Africa and Uganda. The Ugandan studies were mainly on war-affected adolescents [17] and AIDS orphaned children [18]. There were no studies from Burkina Faso. According to the Lifecycle approach (figure 3), good evidence-based problem identification, treatment and care for mental disorders in childhood is believed to have an impact for the individual’s well-being and productivity later in life as well as be a protective factor for the offspring. If the burden of mental disease is reduced due to the early breastfeeding intervention this could increase productivity by reducing psycho-social stressors.

Figure 3: The lifecycle approach to risk factors for mental disorders

*Kieling C, Baker-Henningham H, Belfer M, Conti G, Ertem I, et al. (2011) Child and adolescent mental health worldwide: evidence for action. Lancet 378: 1515-1525.*


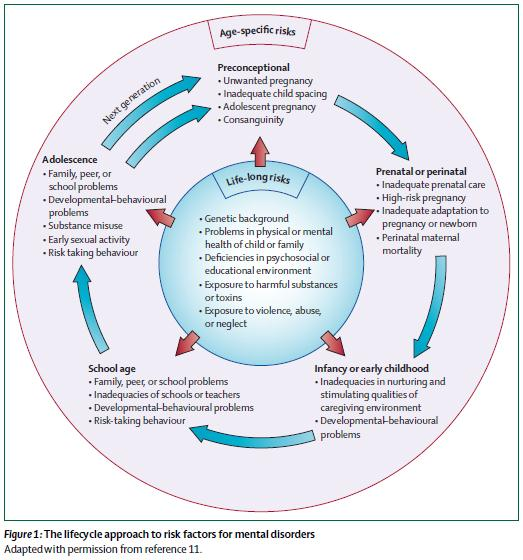


**2.1 STUDY JUSTIFICATION**

The proposed PROMISE-SB will provide information on the extent to which peer counselling for EBF in the first six months of life will influence human capital formation in communities similar to those studied in the PROMISE-EBF trial in Uganda and Burkina Faso. This information is needed from African contexts as most data on this subject are from high-income settings. The study will also provide valuable information on mental health and human capital in our fairly representative population, informing the work towards improved child mental health and improved human capital that will benefit the entire life cycle [15] and thereby country development.

**2.2 AIMS AND OBJECTIVES Overall aim**

To evaluate the effect of peer counselling for EBF in the first six months of life on human capital

formation among children recruited into the PROMISE-EBF trial in Burkina Faso and Uganda.

**Primary objective**

To measure the effect of peer counselling for EBF in the first six months of life on human capital

formation including:

a. cognitive function

b. behavioural and emotional status

c. educational readiness and attainment d. health status

e. fine and gross motor skills

f. physical growth and body composition and g. changes in household socioeconomic status.

**Secondary objective**

To identify solutions to potential barriers that limit scale up for peer counselling for EBF in the

first six months of life

**3.0 STUDY PROCEDURES**

**Overview**

This study will examine the association between promotion of peer counselling for EBF in the

first 6 months of life and human capital including: 1) cognitive function; 2) behavioural and emotional status; 3) educational readiness and attainment; 4) health status; 5) fine and gross motor skills; 6) physical growth and body composition; and 7) changes in household economic status. Below we present items 1, 2, 3 under **mental health;** 4, 5 and 6 under **somatic health** and lastly 7 under **productivity**. We will also assess moderators and confounding factors.

The PROMISE-EBF trial was a community-based, cluster-randomized trial promoting peer counselling for exclusive breastfeeding (EBF) in the intervention group. Peer counsellors living in the intervention clusters were identified and trained for one week. The course material was based on the WHO courses: Breastfeeding counselling: a training course, and HIV and infant feeding counselling: a training course [19, 20]. The courses were integrated and adapted to local circumstances. The counsellors offered home-based breastfeeding peer support to the mothers in the intervention clusters. Peer counsellors received continuous supervision (fortnightly to monthly) by study-appointed supervisors. At least five home visits were scheduled for all women in the study starting with a visit in the third trimester of pregnancy. Peer counsellors provided information, and encouraged and supported EBF for 6 months. Mothers with any breastfeeding difficulties were referred to a health worker with training in lactation management. The control group received the standard of care within the respective countries [1].

**Sites and sampling procedures**

The original study (the PROMISE-EBF trial) conducted from 2006 to 2008 [1] took place in

Uganda, Burkina Faso, Zambia and South Africa. The current study will be conducted in Burkina Faso and Uganda because this is where the intervention had the largest documented impact. In Burkina Faso, the study site was located in rural Banfora, southwest of Burkina Faso. The Ugandan site was Mbale District, including urban Mbale Municipality and adjacent rural areas in Eastern Uganda. Clusters rather than individual mother-infant pairs were randomized to intervention or control to avoid potential spill-over between intervention and control arms and to improve the potential for scaling up this programme-relevant intervention. Clusters were mapped based on criteria of accessibility, population size, and health system characteristics and subsequently randomized by a central coordinating team into intervention or control arms. In total, 24 clusters (12 intervention and 12 control), with an average population size of 1000 inhabitants were selected in each country. In Burkina Faso and Uganda, all pregnant women in the study communities were approached for study participation and 99% consented to be screened for the study. In Uganda randomization was stratified on rural /urban residence reflecting the Uganda society: 20% in the urban and 80% in the rural areas. Overall, 895 pregnant women in Burkina Faso and 863 in Uganda were enrolled in the initial PROMISE-EBF trial. A recruitment interview was scheduled in the antenatal period or latest within one week after birth. Data collection visits were then scheduled at 3, 6, 12 and 24 weeks after birth. Inclusion in the study was a two-stage process involving first the pregnant woman (pre-inclusion) and then the infant (inclusion). Pregnant women intending to breastfeed were considered for study participation. The pre-inclusion criteria were that the woman resided in the selected cluster; was 7 months or visibly pregnant; had no plans to move in the forthcoming year; and provided informed consent. At the 3 week post-partum assessment a pre-included mother-infant pair was included if it was a single birth with no severe malformation which could interfere with breastfeeding. Exclusion criteria included severe psychological illness in the mother which could interfere with consent and study collaboration and intention to replacement feed. Unless a clear reason for non-participation in a scheduled visit was given, three attempts to see the mother-infant pair were made before a visit was considered missed. A recruited mother was revisited until the last scheduled visit irrespective of the number of missed visits; unless there was a clear reason for termination (e.g. declined further consent or moved). Data collection was done by trained independent data collectors in the mothers‟ homes. The data collectors were to the extent possible kept unaware of cluster allocation and were kept separate from peer counsellors and attended home visits on different days to peer counsellors to reduce information bias.

**Follow up procedures**

We anticipate locating and re-enrolling a sizable proportion of the PROMISE-EBF cohort in

Burkina Faso and Uganda. Though the scheduled follow-up of children in the EBF-trial was up to

6 months, follow-up studies have been carried out and published from Burkina Faso at 12 months of age and in Uganda at 2 years of age. These follow up studies had very good overall follow up rates of over 70%, just like the original study (figure 4). A recently concluded study followed up the Ugandan cohort between age 3 and 4 years. The very high follow-up rates were achieved because of the involvement of members in the local communities during data collection. We used recruiters from the clusters to identify the pregnant women. The same recruiters were involved in the 12 and 18 months as well as the 3-4 year follow-up studies in Burkina Faso and Uganda. We also have access to Global Positioning System (GPS) data for the homesteads. Based on previous follow-up data from the PROMISE-EBF study and its subsequent cohort studies, our teams in Centre MURAZ (Burkina Faso) and in Mbale District (Uganda) have different source data and qualified personnel to retrieve a high proportion of children initially enrolled, for their re- inclusion in the PROMISE-SB study. The following useful information and source data are locally available in each of the sites:

1) Full names and dates of birth of all children included in the PROMISE-EBF trial and its subsequent cohort studies per village.

2) Full names of the parents of each child (including nick names when relevant)

3) List of the GPS-coordinates of the households involved in the PROMISE-EBF trial.

4) Members of staff from the PROMISE-EBF study are still in place and can be re- contracted to take part in the PROMISE-SB study and they will be an asset for our teams in retrieving the PROMISE-EBF children. Key personnel who are still available include study coordinators, recruiters, peer-counsellors and data collectors.

We estimate that within a period of 3 months our teams will be able to retrieve over 70% of children initially enrolled and who are still alive. We will request for re-consent for inclusion in the PROMISE-SB study. Data collection is expected to last 12 months.

Figure 4: Follow up in the original study (A=Burkina Faso, B=Uganda)


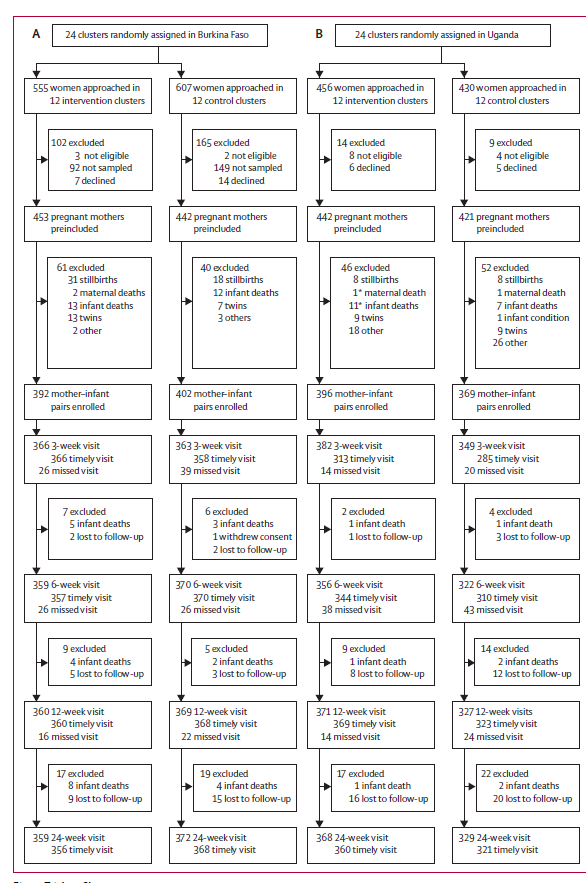


**Assessments**

The assessments will be done through three steps: 1) a home-session which will involve obtaining

an informed consent and parent interviews using structured questionnaires, 2) a clinical and psychometric assessment session of the child, and 3) teacher structured interviews when applicable. The home sessions and clinical sessions will be on different days with the home session coming first.

A home-session will involve assessment of the household status including confirmation that the child is still alive. In the event of a child death, an appointment will be sought for a verbal autopsy.

For live children with informed consent, the home-session, estimated to last about 1-2 hours will include the following: 1) Updates of the socio-demographic information of the household including food security, 2) morbidity (history and current status) of the child, 3) history of intentional and unintentional injuries of the child and caretaker, 4) productivity including schooling and work related activities of the household members, 4) moderators/confounders and

5) parental structured interviews on own and child’s mental health and related factors

The assessment of each child will be done in a group setting, avoiding a single child and single investigator interaction which would be perceived as in an unfamiliar and strange environmental situation. We aim to mimic a ‘school’ situation but, importantly, the children will be scored individually. This group setting is believed to be more child and family friendly, because studies in low-income countries have shown that children in these areas perform better when in the company of other children [21]. A play area within the test sites will be set up for this purpose.

A clinical child assessment will include the following, estimated to take approximately 2-3 hours: medical and developmental history, physical and neurological exam, fine and gross motor skills assessment, anthropometry measures, audiology and visual acuity testing, cognitive testing, laboratory tests: including minimum haemoglobin, CRP and malaria screening. The children and their mothers/guardians will be allowed breaks in between the assessments, and the study team will provide a meal before start-up of the assessment. Any identified illnesses will be referred to the appropriate local or regional health service. When possible, appropriate services will be provided in collaboration with the local health system and NGOs during the period of data collection, for example ophthalmologist and glasses for visual problems, malaria treatment or referral for HIV testing.

**Somatic health**

*Mortality*

The PROMISE-EBF trial has collected and published data on perinatal, neonatal and infant mortality in Burkina Faso and Uganda. For this study, we will collect data on child deaths after infancy. Information on all deaths will be collected and classified using a standard World Health Organization (WHO) verbal autopsy questionnaire that has been validated in both countries and used by our team [8].

*Morbidity*

Using standardized questionnaires, the original PROMISE-EBF trial and subsequent follow-up studies have collected data on immunization, hospitalizations, acute infections (malaria, diarrhea, cough and fever), intentional and unintentional injuries. We will conduct repeated data collection on these topics as the children now are older.

**Clinical examination**

*Clinical and neurological assessment*

A doctor with assistant nurses will review the medical history including the *10 Questions plus* [22] and conduct a physical and neurological exam. Heart rate, blood pressure and body temperature measurements will be recorded. The diagnostic screening of motor disabilities will be assessed according to a structured neurological assessment covering: mental status, cranial nerves, reflexes, motor system: tone; strength/power; gait; coordination and sensation. Diagnosis of cerebral palsy, post-polio paralysis, limb abnormalities and other severe medical conditions will be made on the basis of the medical and neurological assessments. The study will refer children with medical conditions to the health facilities.

*Anthropometry*

Weight will be measured using standardized Seca® digital scales to the nearest 0.1kg. We will use a Seca stadiometer® in the 263 series, to measure height. Anthropometric indices will be calculated assessing weight-for-height, height-for-age, weight-for-age and body mass index (BMI)-for-age z-scores. To measure body composition, mid-upper arm circumference (MUAC), triceps skinfold and subscapular skinfold will be measured. Head circumference will also be measured. For the above measurements z-scores will be calculated using WHO Child Growth Reference 2007 [(www.who.int/childgrowth/en](http://www.who.int/childgrowth/en)). Standardization to minimize technical error of measurement (TEM) will include regular refresher training, assessing precision and reliability of data collectors.

*Fine and gross motor skills*

We will use *The movement assessment battery for children* (Movement ABC or M ABC) consisting of 8 items addressing manual and ball skills and balance (table 4) [23]. Total standard scores and age-specific norms and standard deviations will be presented.

able 4: Test items form age band 3 of the Movement ABC


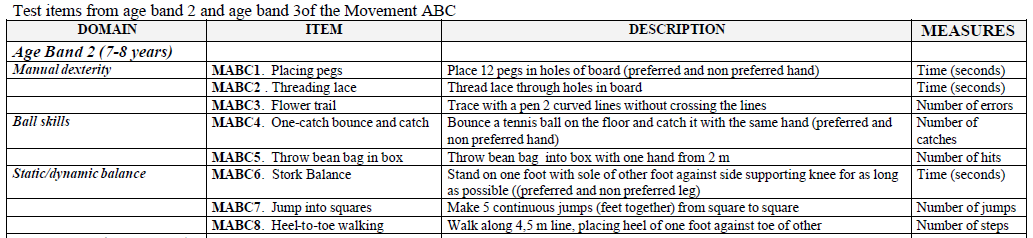


*Visual acuity*

Visual acuity will be assessed using the “Illiterate E chart” (Snellen E chart). This chart does not depend on literacy or familiarity with an alphabet. It is therefore effective in assessing children with cognitive and developmental disabilities who may have higher rates of visual problems than others [24,25]. The diagnostic assessment of vision impairment and eye disease will be made by qualified personnel and the diagnostic criteria will be in accordance with World Health Organization criteria on vision impairments.

*Auditory*

A clinician will assess hearing using otoacoustic emissions (OAEs), tympanometry and conventional and/or conditioned play audiometry (CPA) adapted from Berg and colleagues [26]. To assess middle ear status, we will use low-frequency (220 or 226 Hz) tympanometry. We will determine the type and degree of hearing impairment based on the WHO criteria, 2002. The study team will be trained in the use of OAEs, tympanometer, and audiometer before the study begins.

*Nutritional status*

Having been a nutritional intervention, we have an extensive database of feeding data. Using food frequency questionnaires, data on feeding history was collected at 3, 6, 12 and 24 weeks of age. Data on complementary feeding was also collected at 18 months and 3-4 years of age in Uganda and at 1 year of age in Burkina-Faso. For this study, we shall collect data on food frequency in the past 7 days using food frequency questionnaires that we have already used in our population. Data on food security will be collected based on the food and agricultural organization standards. As mentioned earlier, haemoglobin levels will also be measured.

*Laboratory assessment*

Finger prick screening will be done to assess haemoglobin status, C-reactive protein and malaria parasitaemia. Children with abnormal laboratory findings indicating illness or deficiency results will be referred for further investigation. After careful consideration, we have decided not to include HIV-testing, but only refer for counseling and testing where clinical suspicion warrants it. The reasons are that 1) few children are likely to be infected (in Uganda the HIV-prevalence is

0.7% among children (maximum 4-5 children in our cohort) and in Burkina the prevalence among adults is around 1% and therefore much less in children; 2) infected children born 5-7 years ago are likely to be either deceased or symptomatic; 3) any undiagnosed, asymptomatic HIV- infections is unlikely to measurably bias the exposure-outcome association of interest because of the low prevalence (1); and 4) having an HIV-test in the study commits a lot of resources with risk of deferring families and children from the study.

**Mental health**

*Cognition and memory*

The team will use the Kaufman Assessment Battery for Children (K-ABC), the Luria-version which is to a large extent overlapping, but less language-dependent than the newer version KABC-II from 2004. Kaufman Assessment Battery for Children (K-ABC) was used and reliability checked for these contexts [27]. As a supplement Raven Progressive Matrices as a measure of intellectual function will be used. This has also been extensively used in Africa [28].

*Behavioural and emotional factors*

This study will apply the relatively short Strengths & Difficulties Questionnaire (SDQ) ([http://www.sdqinfo.com/)](http://www.sdqinfo.com/) for a gross assessment of behavioural and emotional factors. This is a screening tool with 25 items on 5 scales. When indicated, the child will be referred for further assessment to appropriate authorities. When applicable, we will administer the SDQ for teachers.

*Mental health co-morbidity assessment*

If any of the SDQ-scorings or home structured interviews and the assessment of the child give an indication for serious mental health challenges for the child, the study team will refer the child for follow-up investigation for potential diagnosis of: inattention and impulsiveness, autism-spectrum disorders, anxiety, depression, compulsive disorders, Tourette/tics, trauma and attachment difficulties. Counseling, therapy and treatment will be covered by the study team when feasible. Consent for information on diagnostic conclusions will be sought for.

**Productivity**

*Socio-economic status*

Household socio-economic status will be measured using structured questionnaires measuring monthly household income and expenditures on health, education, clothing, food, housing and social activities. Data will also be collected on possession of assets such as televisions, radio, mobile phone, chair, cupboard, refrigerator, type of toilet, type of house walls, electricity and water source in the home and others. The Unmet basic needs index tool Core Welfare Indicator Questionnaire Survey (World Bank/Unicef) (CWIQ) [29] will be the basis for scoring poverty on the above listed factors.

*Demographic characteristics*

The PROMISE-EBF database already has multiple demographic characteristics including the age and date of birth for all the children. Data on age and sex will be reported for all family members as well as information on education.

*School involvement*

Each child’s school involvement will be assessed using information collected on the number of months enrolled in school and number of days attended in the past month/year. Children not yet in school will be assessed for school readiness based on findings from somatic and mental health examination.

**Moderators - Social emotional**

The mother’s or caretaker’s wellbeing will be addressed using a survey of health related quality

of life. In order to assess the environment in which the study children are being brought up and the relationship with the caregiver, a moderated African validated version of the HOME Screening Questionnaire will be used [30].

*Quality of relationships*

Caregivers will also be aided to complete the 34-item short form of The Parent Child Relationship

Inventory (PCRI) [135] in order for us to assess the caregiver-child relationship.

The PCRI yields four validated scores: 1) involvement; 2) quality of communication; 3) limit setting; and 4) autonomy.

*Relationships with peers*

These will be assessed using the Strengths and Difficulties Questionnaire (SDQ) as discussed in the behavioural and emotional sections above.

*Caregiver’s mental health and stress*

Caregivers will be requested and aided to complete the following interviews 1) the World Health Organization SRQ-20 [31], a self-reporting tool designed to assess the intensity of depressive symptoms experienced in the past 4 weeks; and 2) the Depression, Post-Traumatic Stress Disorder, and Substance Abuse modules from the Clinical Diagnostic Questionnaire (CDQ).

*Community*

The study group will address social networking, access to services and not-income generating activities and interests by identifying other actors in the study communities that might interfere with our exposure-outcome of interest.

We will be working in two low-income countries where resources are scarce, including stable power, clean water and sanitation, infrastructure and networking. We will assess around 800 child-mother (care-taker) pairs in their home-setting and a nearby facility in each country. It is evident that high-tech assessment, such as MRI, laboratory tests, etc., is not possible within the budget. Table 5 below shows maximum and minimum estimates of time for the different activities and assessments.

**Implementation**

As earlier mentioned, data collection will involve three steps: 1) a home-session, 2) clinical and

psychometric assessment session of the child, and 3) teacher structured interviews (when)

applicable.

*The home-session*

This will to a large extent be done by our data-collectors from the PROMISE EBF study. They will visit the households in the clusters assigned during the original data capturing. They will obtain informed consent and conduct most of the structured interviews addressed to the caretaker.

*Clinical and psychometric assessment session*

We will establish a number of study locations where we ensure a similar setup of our equipment. The equipment will move from one location to another – so we will use the same staff for the entire study population at each site. We imagine that a number of children from the same clusters will travel with one care-taker each to the study location work from test-to-test. The study locations will be school-buildings, administrative buildings or similar. We will try to avoid using health units knowing that many children have bad memories from those places. We will aim at making the place look safe and comforting.

Table 5: Maximum and minimum estimates of time for the different activities and assessments

| Activity |  | Estimated | Estimated |
| --- | --- | --- | --- |
|  |  | minimal time | maximal time |
|  |  | (minutes) | (minutes) |
| Home session | Introduction | 5 | 10 |
|  | Informed consent | 5 | 15 |
|  | Socio-demographic | 5 | 15 |
|  | Morbidity/hospitalization | 2 | 15 |
|  | Injuries | 2 | 15 |
|  | Productivity | 2 | 15 |
|  | Moderators/confounders | 10 | 20 |
|  | HOME-moderated | 5 | 10 |
|  | PCRI | 15 | 15 |
|  | SRQ-20 | 5 | 10 |
|  | SDQ | 10 | 20 |
|  | Closing/appointments | 5 | 10 |
| Summary, home |  | 71 | 170 |
|  |  |  |  |
| Assessment child | History, including 10 questions + | 5 | 10 |
|  | Clinical exam | 10 | 20 |
|  | Motor function | 20 | 40 |
|  | Anthropometry | 5 | 10 |
|  | Audiology | 5 | 15 |
|  | Vision | 5 | 15 |
|  | Cognitive function | 35 | 70 |
|  | Laboratory tests | 5 | 10 |
|  | Closing/appointments/referral | 10 | 20 |
| Summary, clinical |  | 95 | 200 |
|  |  |  |  |
| School contact | Teacher SDQ | 10 | 20 |
|  | Logistics/school | 5 | 10 |

**4.0 DATA COLLECTION AND MANAGEMENT**

Data will be collected on paper CRFs that will be entered in the study database for each site.

Previously collected data, as required, will be retrieved from the PROMISE EBF database.

**Statistical analysis**

The main exposure variable in this study will be having been randomized to an EBF peer

counselling cluster in the first six months of life. The main outcomes will be cognitive function and behavioural and emotional status, schooling, physical health and productivity. Analysis will be country specific and adjusted for clustering and site stratification.

We will estimate the association between the exposure and each of the continuous outcomes using linear regression. All these analyses will then be adjusted for baseline differences between groups (potential sources of confounding) in the original study such as socio-economic status and maternal education, in a multivariable linear regression. For categorical outcomes (e.g. food security) differences between the exposed and unexposed groups will be examined using chi- square tests. Prevalence ratios will be estimated using generalized linear models for the binomial family with a log link. Potential confounders will be adjusted for using multivariable regression models. All regression analyses will take into account the design effect (given the clustered nature of the original study design) and stratification.

The study team has established work packages for our secondary outcomes including scale-up, business packages and marketing of our intervention. Qualitative data for the secondary outcome on identifying solutions to potential barriers that limit scale up for peer counseling for EBF in the first six months of life was collected in the original study but not completely analyzed [32,33]. In this round, this data will be completely analyzed to meet this objective. To address the second secondary objective, health economists are already involved in the PROMISE EBF study and will address these issues in depth [34].

**5.0 ETHICAL CONSIDERATIONS Ethical review**

The study protocol will be submitted to and approved by the relevant Ethics Committees of the

two study sites and from the Institutional Review Board at the Centre for International Health, University of Bergen when requested. In Uganda, approval to conduct this study will be sought from Makerere University College of Health Sciences School of Medicine Research and Ethics Committee (SOMREC) and Uganda National Council of Science and Technology (UNCST).

**Informed consent**

Written informed consent for participation in the main study will be obtained from the mother of

a participating mother-child pair, or a legal caretaker in the event that the mother is deceased or cannot be traced through reasonable effort. (Appendix II). The informed consent procedure will be implemented with an independent third-party (witness) chosen by the mother/ legal caretaker for illiterate mothers/caretakers. This witness will sign the consent form with the mother/caregiver and research assistant.

In the Uganda site, additional consent will be obtained from parent/guardians of children identified to have serious mental health challenges who will require additional investigation to improve diagnostic accuracy such as magnetic resonance imaging (MRI).

**Risk and Benefits**

*Risks*

There are no anticipated serious risks to children involved in this study. Children may experience slight discomfort, pain or bruising during blood sampling. However, all attempts will be made to minimize this. To the best of our knowledge, there is no anticipated risk associated with conducting an MRI on children for whom this test will be done.

*Benefits*

Through extensive assessments, this study will identify children with abnormal laboratory findings indicating illness or deficiency as well as other medical conditions. Such children will be appropriately referred for further investigation and care.

*Compensation*

All children will be given a meal at the start of the day and a snack such as fruits and water during the breaks. Participants will be provided transport refund where applicable.

**5.1 INSTITUTIONAL/ORGANIZATIONAL AND MANAGEMENT CAPACITY Consortium description**

The PROMISE Saving Brains research group is a subgroup within the PROMISE research

consortium. The PROMISE Saving Brains consists of three universities and research institutions in Africa and Europe with the aim to conduct long-term follow-up studies of the PROMISE EBF study in Uganda and Burkina Faso. The three institutions are:

• Makerere University, Uganda

• Centre Muraz, Burkina Faso which is a publicly funded health research institution

• University of Bergen, Norway

**Makerere University, Uganda**

The study will be coordinated by the Department of Paediatrics and Child Health at Makerere

University. The Department is part of the School of Medicine which in turn is part of the College of Health Sciences. The Department was involved in the original European Union (EU)-funded PROMISE-EBF study, successfully producing the required financial reports for EU. The University, founded in 1922, is fully capable of running internationally funded research projects at all levels.

**Centre Muraz, Burkina Faso**

Centre Muraz is a publicly funded health research institution, owned by the Ministry of Health.

The Centre was involved in the original EU-funded PROMISE-EBF study, successfully

producing the required financial reports for EU. Centre Muraz, founded in 1939, is fully capable of running internationally funded research projects at all levels.

**University of Bergen, Norway**

The unit at the university that will be involved in the study is the Centre for International Health.

The Centre was the principal coordinator of the original PROMISE-EBF study, including the administration of the EU funds. The University is fully capable of running internationally funded research projects at all levels.

**Institutional management and staffing plan**

*Overall study management*

The PROMISE Saving Brains will be guided by a Steering Committee (SC) of six which will include the PI, Co-PI and at least one Investigator from each of the following: Norway, Burkina Faso and Uganda.

One person from each institution is enough for a quorum. The Principal Investigator is the chairperson and has a casting vote.

The SC will be responsible for the conduct and coordination of the study. The SC is the decision making body for all scientific and administrative aspects. It will send reports to the funding agency, ethical committees and regulatory bodies. SC participants will meet on regular conference calls and face-to-face annually.

*Site coordination*

In Uganda and Burkina Faso there will be a Site Steering Committee (SSC), consisting of all investigators of each site and the study coordinator, led by the site PI. It will plan local activities, solve local problems and report to the SC. In each site, a study coordinator will be in charge of general coordination of the study on site. (S)he will be in charge of the day-to-day conduct of the study and reports to the national PI.

*Community Liaison Group*

A community liaison group (CLG) will be established in each site. CLG members will meet quarterly.

This group will be made up of volunteer members from the community. The CLG will act as a communication structure between the community and the investigators to give feedback on participant concerns and to communicate key messages about the study. In addition, they will provide advice and insights to the investigators regarding community attitudes, perceptions and behaviours that might influence the implementation or outcome of the study.

*Staffing plan*

In each site, the project will employ the following: a study coordinator, study doctors for clinical assessment, neuropsychologists for psychometric testing, data collectors for parent interviews and obtaining consent, a data manager, research assistants and a driver.

**Study Timing**

This is detailed under the Project Framework (Appendix I)


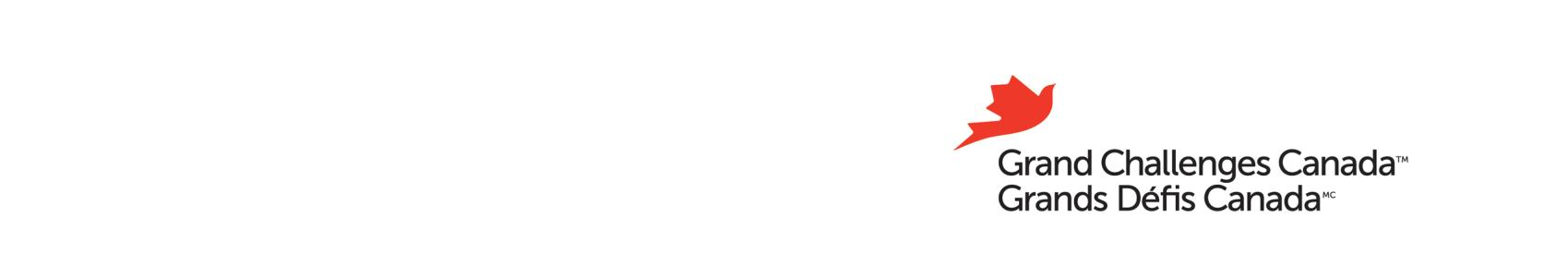


**APPENDIX I PROJECT FRAMEWORK TABLE**

| **Objectives** | **Activities** | **Critical milestones / Metrics of success** | **Key risks** | **Mitigation strategies** | **Estimated dates** |
| --- | --- | --- | --- | --- | --- |
| **Objective 1:**  To estimate the effect of peer counseling for exclusive  breastfeeding (EBF) in the first six months of  life on human capital  formation | **Activity 1.1:** Preparation for study implementation | **Proof of ethics approval from School of Medicine Research and Ethics Committee, Uganda**  **National Council for Science and Technology, Centre**  **MURAZ Ethics Review**  **Committee, and Burkina Faso National Health Research Ethics Committee submitted to Grand Challenges Canada** (September 2012)  Staff trained  Community mobilization workshops held  **Submit plan to incorporate finalized Saving Brains core outcome metrics for approval by Grand Challenges Canada** (December 2012)  Pilot completed that | Delay in ethical approval  Attrition of trained staff  Tools might be misunderstood | Submit protocol to IRB  on time  Train „reserve staff‟  Modify tools after pilot testing | July- September  2012 |


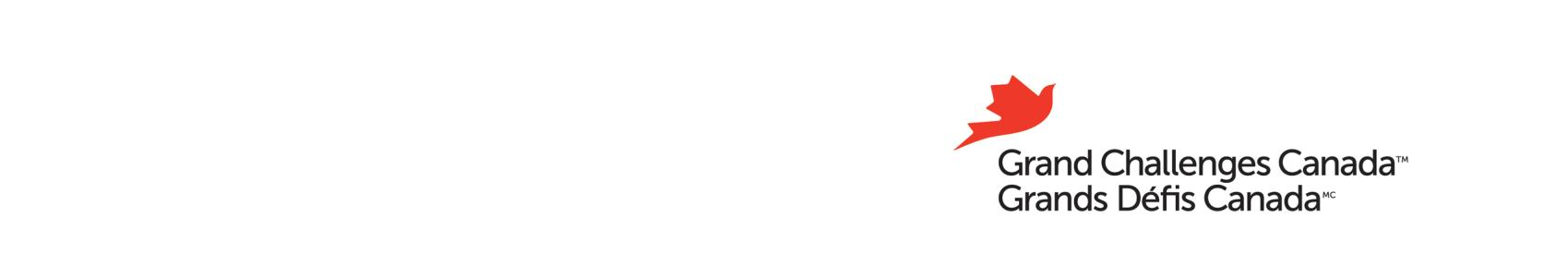


|  |  | demonstrates successful adaptation / modification of tools  **Submit inclusion plan for selecting participants for MRI to Grand Challenges Canada** (September 2012) |  |  |  |
| --- | --- | --- | --- | --- | --- |
|  | **Activity 1.2**  Establish standard operating procedures  (SOP) for the project | SOP documents (finance, scientific, management) complete. | Delay in finalizing the SOPs | Start developing SOPs even before IRB approval is obtained | August- September  2012 |
|  | **Activity 1.3**: Establish an efficient information and communication system | Communication strategy document.  Functional „Skype‟ and other  IT communication systems.  Minutes of online meetings  Functional website. | Power outages | Practical alternative power source | July-October  2012 |


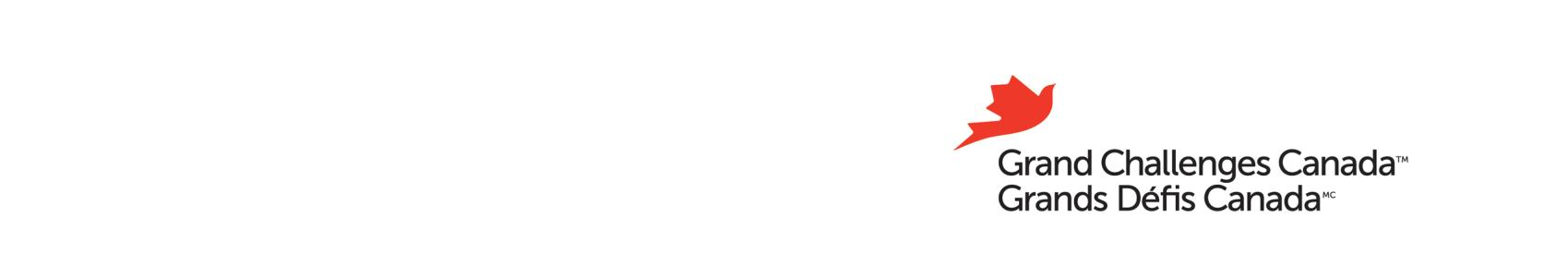


|  | **Activity 1.4 :**  Enroll participants | **Minimum of 428 (404 + 6% attrition) of original EBF intervention and control groups enrolled in Uganda traced and enrolled;**  **selection biases are identified and randomization integrity confirmed.**  **Minimum of 428 (404 + 6% attrition) of original EBF intervention and control groups in Burkina Faso traced and enrolled;**  **selection biases are identified and randomization integrity**  **confirmed.**  Indicator of success: 70% (604+626) of original EBF intervention and control groups enrolled in each site. | Loss to follow up | Use GPS coordinates and other information to trace participants | October  2012-March  2013 |
| --- | --- | --- | --- | --- | --- |
|  | **Activity 1.5:** Assess health, nutrition and socioeconomic (SE) status | Community level confounders captured and assessed.  Data collection complete | Recall and information bias  Technical faults with software and computers | Use of community based data collectors  Data backups  Use available records  Technical support staff | March 2013- May 2014 |
|  | **Activity 1.6**  Assessment of  neurodevelopment, cognition, behavior / | **Assessments completed on**  **94% of enrolled participants tracked to date in Uganda**  **(June 2013).** | Information bias | Blinding of data collectors as to which group received peer counseling* | April 2013- May 2014 |


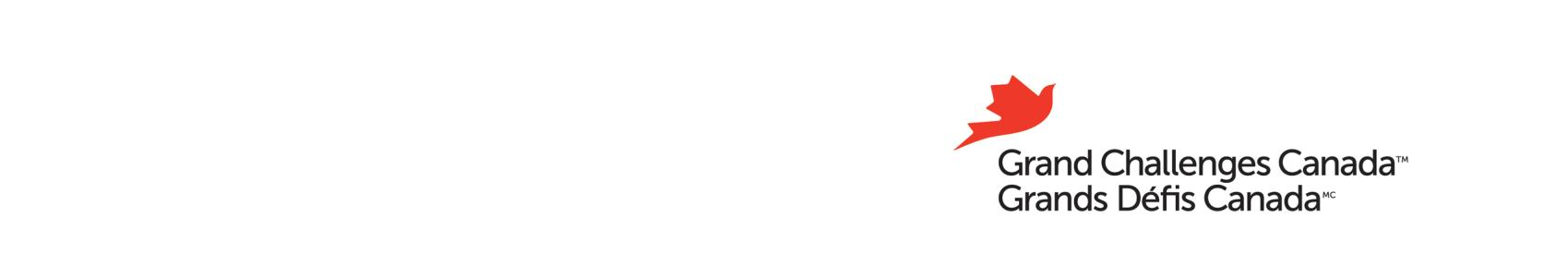


|  | emotion and school readiness | **Assessments completed on**  **94% of enrolled participants tracked to date in Burkina Faso (June 2013).**  Assessments complete on minimum of 404 of original EBF intervention and control groups in each site (Uganda and Burkina Faso) with comparability of scores validated on a random 10% of double-scored assessments from two assessors.  Indicator of success: 70% (604 in Uganda, 626 in Burkina Faso) of original EBF intervention and control  groups enrolled in each site. |  |  |  |
| --- | --- | --- | --- | --- | --- |
|  | **Activity 1.7**  Neuroimaging of a  subset of Ugandan participants | MRI analysis is conducted according to the inclusion plan. | Guardians or parents of children with neurocognitive abnormalities might refuse neuroimaging  MRI machine could break down  Loss of MRI data | Give detailed information about the study especially the need and safety of MRI. Separate consent for MRI  Arrange for alternative MRI (Kololo and Mulago Hospitals)  Back up copy of images | April 2013- May 2014 |


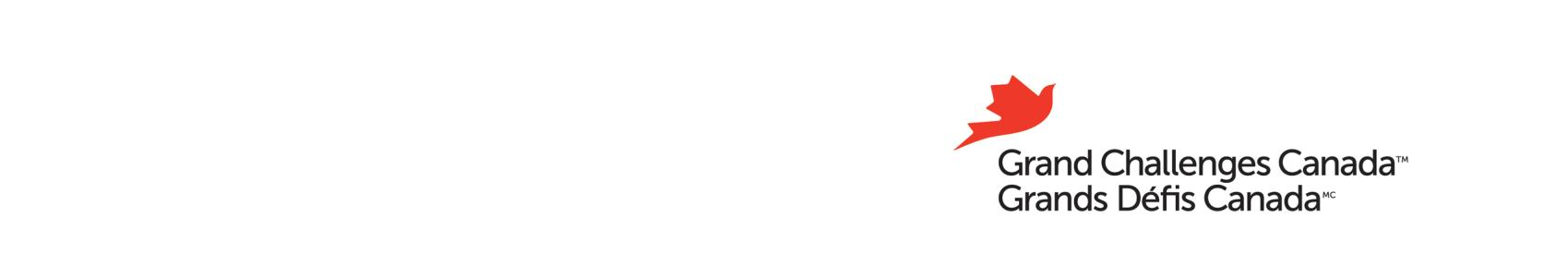


|  |  |  |  | on CDs and keep them in  2 separate locations |  |
| --- | --- | --- | --- | --- | --- |
|  | **Activity 1.8**  Analysis of data | Report on:  - Health, nutrition and SE  status.  - Neurodevelopment, cognition, behaviour and  school readiness.  Final paper on effect of peer counseling for EBF on neurodevelopment, cognition, behaviour and school readiness. | Loss of data set  Delay in data analysis and manuscript writing | Back up data  Update anti-virus guard  Timely data entry and cleaning | March-June  2014 |
| **Objective 2:** To identify solutions to barriers that limit scale up for peer counseling (PC) for EBF | **Activity 2.1**  Collection of data on barriers, facilitators  and solutions to scale up | Initial path-to-scale submitted to Grand Challenges Canada (January 2013)  Recommendation report on barriers, facilitators and solutions to peer counseling for EBF | Participants may tell us what they think is the ideal response | Training of research team on qualitative research methods | May- November  2013 |
|  | **Activity 2.2**  Policy analysis on potential for integrating PC in the  health care system | Recommendation for policy report on peer support for EBF. | Failure to access unpublished literature | Collaboration with Ministry of Health, NGO‟s and other stakeholders | November  2013-June  2014 |
|  | **Activity 2.3**  Develop cost effective delivery models | Reanalysis of previous EBF data on acceptability and intensity of peer counseling completed. | Challenges in managing large data sets | Data backups  Technical and statistical support staff | October  2013-April  2014 |


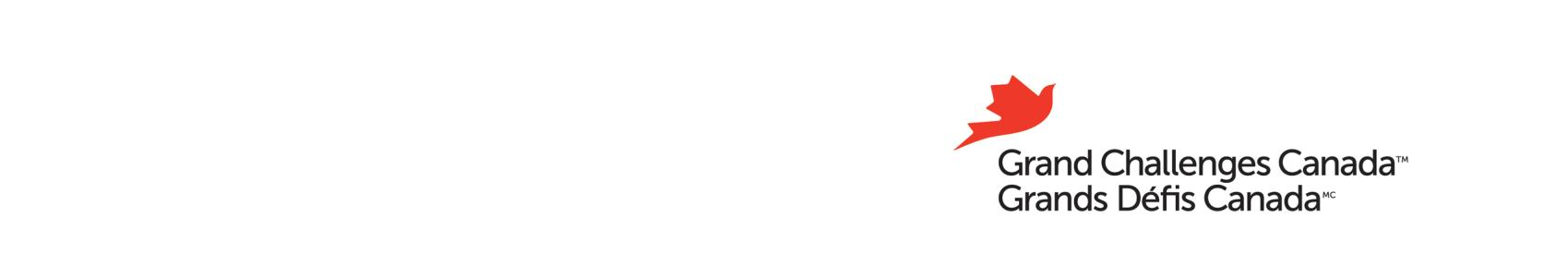


|  |  | Cost effective delivery model report |  |  |  |
| --- | --- | --- | --- | --- | --- |
| **Objective 3:** To disseminate findings | **Activity 3.1**  Develop dissemination plan | Dissemination plan submitted to Grand Challenges Canada (June 2013) |  |  | March-June  2013 |
|  | **Activity 3.2**  Organize scientific  dissemination meetings | At least three dissemination meetings held  Reports of scientific meetings | Disruption by cultural events | Plan meetings outside cultural events such as circumcision ceremony | September  2013-June  2014 |
| **Community- wide Milestones** | Execute Saving Brains Data Access Agreement containing at least the following key terms (September  2012):  • Data resulting from your Grand Challenges Canada grant and previously generated data required to fulfill the objectives of your Grand Challenges Canada grant will be made available to Saving  Brains Economics program Grantees in furtherance of Global Access.  • The onus is on the Saving Brains Grantee to ensure that consent forms and protocols do not restrict access to data collected as a result of your Grand Challenges Canada grant in a manner inconsistent with the Data Access Agreement and/or the principles of Global Access.  • Access to Data will be provided by the Saving Brains Grantee within 6 months of data cleaning.  • Data will be provided by Saving Brains Grantees cleaned and in a computer readable digitized format. | | | | September  2012 |

**CITATIONS**

1. Tylleskar T, Jackson D, Meda N, Engebretsen IM, Chopra M, et al. (2011) Exclusive breastfeeding promotion by peer counsellors in sub-Saharan Africa (PROMISE- EBF): a cluster-randomised trial. Lancet.

2. Institute-of-Medicine (2001) Neurological, Psychiatric and Developmental Disorders: Meeting the challenge in the developing world: National Academy Press.

3. Brion MJ, Lawlor DA, Matijasevich A, Horta B, Anselmi L, et al. (2011) What are the causal effects of breastfeeding on IQ, obesity and blood pressure? Evidence from comparing high-income with middle-income cohorts. Int J Epidemiol 40: 670-680.

4. (2010) WHO & Mental health and poverty project. Mental health and development: Targeting people with mental health conditions as a vulnerable group. 74.

5. Michaelsen KF, Lauritzen L, Mortensen EL (2009) Effects of breast-feeding on cognitive function. Adv Exp Med Biol 639: 199-215.

6. Daniels MC, Adair LS (2005) Breast-feeding influences cognitive development in

Filipino children. J Nutr 135: 2589-2595.

7. Veena SR, Krishnaveni GV, Srinivasan K, Wills AK, Hill JC, et al. (2010) Infant feeding practice and childhood cognitive performance in South India. Arch Dis Child 95:

347-354.

8. Zarrella KL, Schuerger JM (1990) Temporal stability of occupational interest inventories. Psychol Rep 66: 1067-1074.

9. Anderson JW, Johnstone BM, Remley DT (1999) Breast-feeding and cognitive development: a meta-analysis. Am J Clin Nutr 70: 525-535.

10. Drane DL, Logemann JA (2000) A critical evaluation of the evidence on the association between type of infant feeding and cognitive development. Paediatr Perinat Epidemiol 14: 349-356.

11. Kramer MS, Chalmers B, Hodnett ED, Sevkovskaya Z, Dzikovich I, et al. (2001) Promotion of Breastfeeding Intervention Trial (PROBIT): a randomized trial in the Republic of Belarus. JAMA 285: 413-420.

12. Sacker A, Quigley MA, Kelly YJ (2006) Breastfeeding and developmental delay:

findings from the millennium cohort study. Pediatrics 118: e682-689.

13. Kramer MS, Aboud F, Mironova E, Vanilovich I, Platt RW, et al. (2008) Breastfeeding and child cognitive development: new evidence from a large randomized trial. Arch Gen Psychiatry 65: 578-584.

14. Nagashima K, Itoh K, Kuroume T (1990) Levels of insulin-like growth factor I in full- and preterm human milk in comparison to levels in cow's milk and in milk formulas. Biol Neonate 58: 343-346.

15. Kieling C, Baker-Henningham H, Belfer M, Conti G, Ertem I, et al. (2011) Child and adolescent mental health worldwide: evidence for action. Lancet 378: 1515-1525.

16. Nordhov SM, Kaaresen PI, Ronning JA, Ulvund SE, Dahl LB (2010) A randomized study of the impact of a sensitizing intervention on the child-rearing attitudes of parents of low birth weight preterm infants. Scand J Psychol.

17. Bolton P, Bass J, Betancourt T, Speelman L, Onyango G, et al. (2007) Interventions for depression symptoms among adolescent survivors of war and displacement in northern Uganda: a randomized controlled trial. JAMA 298: 519-527.

18. Kumakech E, Cantor-Graae E, Maling S, Bajunirwe F (2009) Peer-group support intervention improves the psychosocial well-being of AIDS orphans: cluster randomized trial. Soc Sci Med 68: 1038-1043.

19. WHO, Unicef (1993) Breastfeeding counselling: A training course. Geneva.

20. WHO (2005) HIV and infant feeding counselling tools: Reference guide. Geneva.

21. Kashala E, Elgen I, Sommerfelt K, Tylleskar T, Lundervold A (2005) Cognition in

African children with attention-deficit hyperactivity disorder. Pediatr Neurol 33:

357-364.

22. Wu L, Katz J, Mullany LC, Haytmanek E, Khatry SK, et al. (2010) Association between nutritional status and positive childhood disability screening using the ten questions plus tool in Sarlahi, Nepal. J Health Popul Nutr 28: 585-594.

23. Engel-Yeger B, Rosenblum S, Josman N (2010) Movement Assessment Battery for Children (M-ABC): establishing construct validity for Israeli children. Res Dev Disabil 31: 87-96.

24. Mwanza JC, Nkidiaka CM, Kayembe DL, Maillet CY, Mukau EJ, et al. (2000) Ophthalmologic abnormalities in mentally retarded. Bull Soc Belge Ophtalmol: 75-

78.

25. Thulasiraj RD, Nirmalan PK, Ramakrishnan R, Krishnadas R, Manimekalai TK, et al. (2003) Blindness and vision impairment in a rural south Indian population: the Aravind Comprehensive Eye Survey. Ophthalmology 110: 1491-1498.

26. Berg AL, Papri H, Ferdous S, Khan NZ, Durkin MS (2006) Screening methods for childhood hearing impairment in rural Bangladesh. Int J Pediatr Otorhinolaryngol

70: 107-114.

27. Bangirana P, Seggane M, Allebeck P, Giordani B, John CC, et al. (2009) A preliminary examination of the construct validity of the KABC-II in Ugandan children with a history of cerebral malaria. Afr Health Sci 9: 186-192.

28. Raven JC, Court J, Raven J (1990) Manual for Coloured Progressive Matrices: Section 2.

29. (2004) CWIQ Core Welfare Indicators questionnarie. St. Lucia, Barbados: Department of statistics. St. Lucia, Barbados: Department of statistics, UNDP.

30. Frankenburg WK, Coons CE (1986) Home Screening Questionnaire: its validity in assessing home environment. J Pediatr 108: 624-626.

31. (1994) WHO SRQ-20. Geneva.

32. Nankunda J, Tylleskar T, Ndeezi G, Semiyaga N, Tumwine JK (2010) Establishing individual peer counselling for exclusive breastfeeding in Uganda: implications for scaling-up. Matern Child Nutr 6: 53-66.

33. Nankunda J, Tumwine JK, Nankabirwa V, Tylleskar T (2010) "She would sit with me": mothers' experiences of individual peer support for exclusive breastfeeding in Uganda. Int Breastfeed J 5: 16.

34. Chola L, Nkonki L, Kankasa C, Nankunda J, Tumwine J, et al. (2011) Cost of individual peer counselling for the promotion of exclusive breastfeeding in Uganda. Cost Eff Resour Alloc 9: 11.

**APPENDIX II: INFORMED CONSENT FORM**

**PARENT/GUARDIAN PERMISSION FOR A MINOR TO PARTICIPATE IN RESEARCH**

Title of study: **SAVING BRAINS IN UGANDA AND BURKINA FASO (PROMISE SB)**

***Principal Investigator****: Prof James K Tumwine (Makerere University School of Medicine)*

***Co-Investigators****:*

*Ass Prof Grace Ndeezi (Makerere University School of Medicine) Dr Victoria Nankabirwa (Makerere University School of Medicine) Dr Angellina Kakooza (Makerere University School of Medicine)*

**Introduction:**

Your child is invited to participate in a study that will examine the association between promotion

of peer counseling for exclusive breastfeeding (EBF) in the first 6 months of life and cognitive function, behavioural and emotional status, educational readiness and attainment, health status, motor skills, physical growth and changes in household economic status. These will be assessed in children and households who/which previously participated in a community-based study promoting peer counseling for EBF in Mbale. In the previous study, peer counselors offered home-based breastfeeding support to the mothers for 6 months in some communities whereas such peer support was not offered in other communities.

In both communities we will collect information about the socioeconomic status, the health and behaviour of each child eligible to participate in this study as well as perform a physical and mental state assessment.

If you decide to let your child participate in the study, you will be asked to sign and date the end of this form. Do not sign this form unless you understand the information in it and have had your questions answered to your satisfaction. You will be given a copy of the signed form. You should keep your copy for your records as it has information, including important names and telephone numbers, to which you may wish to refer during the study period or later.

Taking part in this research study is entirely your choice. You can decide to stop taking part in this study at any time for any reason.

Please read (or have it read to you) all of the following information carefully as it contains important information about the study. Ask Prof. J. Tumwine, or his representative, to explain any words or sections that are unclear to you. You should also ask any questions that you have about this study.

**WHY ARE WE DOING THIS STUDY?**

The purpose of this study is to find out whether peer support for exclusive breastfeeding during

the first 6 months of life improves cognitive function, behaviour and emotional status, educational readiness and attainment, health status, and physical growth of children.

Many conditions occurring during pregnancy and up to 2 years of age may lead to developmental disorders because they affect children, at a particularly vulnerable stage, slowing or stopping the development of the brain and nervous system. Nutrition, particularly breastfeeding has been recognized as an important determinant of cognitive functioning. Breastfeeding may contribute to brain growth and development through either some constituent of breast milk or through the physical and social interactions associated with breastfeeding or a combination. In our previous study, 8 out of 10 mothers who were supported by peer counselors were likely to exclusively breastfeed their infants compared to 4 out of 10 in communities where there was no peer support. It is possible that the increased numbers of exclusively breastfed babies could result in improved brain development and performance at school and as adults. Most studies in which a positive association between breastfeeding and brain function has been found are from high-income countries. In the current study we are assessing the effect of peer support for exclusive breastfeeding on cognitive function, behaviour and school readiness among children in Uganda.

**Who and how many children will be involved in this study?**

We are carrying out this study on at least 428 children of the original PROMISE EBF cohort from

both the intervention and control groups. These children are currently aged 5 to 7 years and some have started schooling.

**How will the study be done?**

Children and their mothers from the original PROMISE EBF study will be identified using

information that we previously collected and the original community recruiters. Once you allow your child to join this study, we shall ask you questions about your child’s growth, development, and behaviour and school readiness. We shall also collect information about your social status and source of income. You will be requested to bring your child to the nearest health centre for taking the weight, height and a detailed medical examination to assess if your child has any illness or disability. At the health center your child’s eyes, ears, hearing and cognitive function will be assessed. In addition the amount of blood the child has (haemoglobin level) will be measured using a finger prick sample. It may also be necessary to visit your child at school and observe his/her performance and interaction with other children, or talk to the teachers and even examine the child’s progress reports. If your child is suspected to have some abnormality with the brain, a specialized investigation called MRI will be performed in Kampala. We shall update the information about your contacts and address so that we can contact you when need arises.

**What are the possible Risks or discomforts from this study?**

The possible discomforts may arise during physical examination and when collecting blood for

measuring the amount of blood the child has. Most of the tasks performed during the physical examination are acceptable during a standard medical examination and carry no more than minimal risk. Should your child be eligible for further screening tests, additional information will be provided and your consent obtained before further tests are undertaken. The MRI is an investigation that carries minimal risk.

**What are the benefits for you or your child being in this study?**

For the individual child we may be able to detect illnesses or abnormalities that were not

previously known. Such children will receive the appropriate standard of medical care provided by the study team or will be referred for expert care if necessary.

**Confidentiality**

Your child’s identifier information including the study number will not be used in any

publication/ presentations about this study. The study information obtained in the course of this study will not be released to anyone outside the study team without your written permission except to the School of Medicine Research and Ethics Committee (SOMREC), Uganda National Council of Science and Technology (UNCST), and where compelled by courts of law. The study information will be kept under a lockable cabinet in the office of the principal investigator or his assistant.

**Compensation**

**For injury:** If your child suffers any physical harm due to the procedures in this study, the

research team will provide immediate treatment or refer your baby for the most appropriate care. There will be no monetary compensation for incidental or indirect losses.

**For time and inconvenience:** The time you spend on this study will not be compensated. We shall provide transport refund at the public transport service rate and lunch during the appointments for physical examination and cognitive assessment.

**Rights of the patient**

Your child’s participation in this study is voluntary. Whether or not you choose to participate, the

quality of care of your child will not be affected. You have the right to refuse or with draw your child from the study if you wish to do so without any explanations. You also have the right to know the results of the tests done.

**Who to contact for more information or your rights in this study**

If you have questions about this research, need any more information, or if you or your child has a medical problem or is hurt in the study, you should contact the Principal Investigator, Prof James K Tumwine, telephone number 0772494120.

If you have questions about your rights or your child’s rights as a volunteer, you may contact the Makerere University School of Medicine Research and Ethics Committee (SOMREC) through the deputy chair, Dr Imelda Namagembe on telephone number +256 414 533541 (the chair person Prof James Tumwine is the principal investigator on this protocol). Alternatively you can contact the Uganda National Council for Science and Technology (UNCST) on telephone +256 414705500, the organization which oversees the conduct of research throughout Uganda.

**What does your signature mean?**

Your signature or thumb print below means that you have understood and are satisfied with the

explanation given to you about this consent form. If you sign the form, it means that you agree to join the study. You will receive a copy of this consent form.

**PARENT’S STATEMENT**

**Documentation of Permission**

I have been given a copy of this form. I have read it or it has been read to me. I understand the information and have had my questions answered to my satisfaction.

I agree to allow to take part in this study.

Name of child

Signature or thumbprint of Parent/caretaker Date

I have fully explained to (name of parent/caretaker) the nature and purpose of the above described study and the risks that are involved. I have answered all questions to the best of my ability.

Signature of Principal Investigator or Representative Date

Signature of Witness (if mother/caretaker cannot read and write) Date
